# Fourier-based chemometric resolution of the FOLFOX regimen through digital filtering with poly-metric sustainability assessment

**DOI:** 10.1038/s41598-026-67979-9

**Published:** 2026-09-11

**Authors:** Hadir M. Maher, Salma Mahmoud Mohamed, Ekram M. Hassan, Amira Fawzy El-Yazbi

**Affiliations:** Pharmaceutical Analytical Chemistry Department, Faculty of Pharmacy, University of Alexandria, Elmessalah, Alexandria, 21521 Egypt

**Keywords:** FOLFOX regimen, Spectrophotometric method, Simultaneous determination, Oxaliplatin, Chemometrics, Fourier functions, Green-by-design, Whiteness, Blueness, VIGI, Chemistry, Engineering

## Abstract

The FOLFOX regimen, comprising oxaliplatin (OXA), 5-fluorouracil (5-FU), and leucovorin (LU), is a standard first-line protocol for colorectal cancer therapy. Given its clinical centrality, developing rapid and high-throughput analytical tools for simultaneous determination of its components is essential. However, severe spectral overlap among the three drugs severely complicates their direct spectrophotometric analysis. In this study, novel spectrophotometric methods were developed and validated for the concurrent quantification of OXA, 5-FU, and LU without preliminary separation steps. While direct and first-derivative spectrophotometric analysis successfully resolved binary fractions, complete resolution of the ternary mixture was uniquely achieved through a hybrid double divisor ratio spectra (HDDR) approach integrating trigonometric Fourier functions. The methods were validated in accordance with ICH Q2(R1) guidelines, exhibiting high linearity (r > 0.999), excellent recoveries (98.0–102.0%), and high precision (RSD < 2.0%) across therapeutic concentration ranges. Furthermore, multi-metric evaluation tools demonstrated improved scores relative to reported chromatographic methods, including higher AES, AGREE, MoGAPI, AGSA, CaFRI, RGB, BAGI, and VIGI scores. The developed HDDR method offers an eco-friendly, rapid, and cost-effective alternative for routine quality control of the FOLFOX regimen.

**Supplementary Information:**

The online version contains supplementary material available at https://doi.org/10.1038/s41598-026-67979-9.

## Introduction

**Fig. 1:**
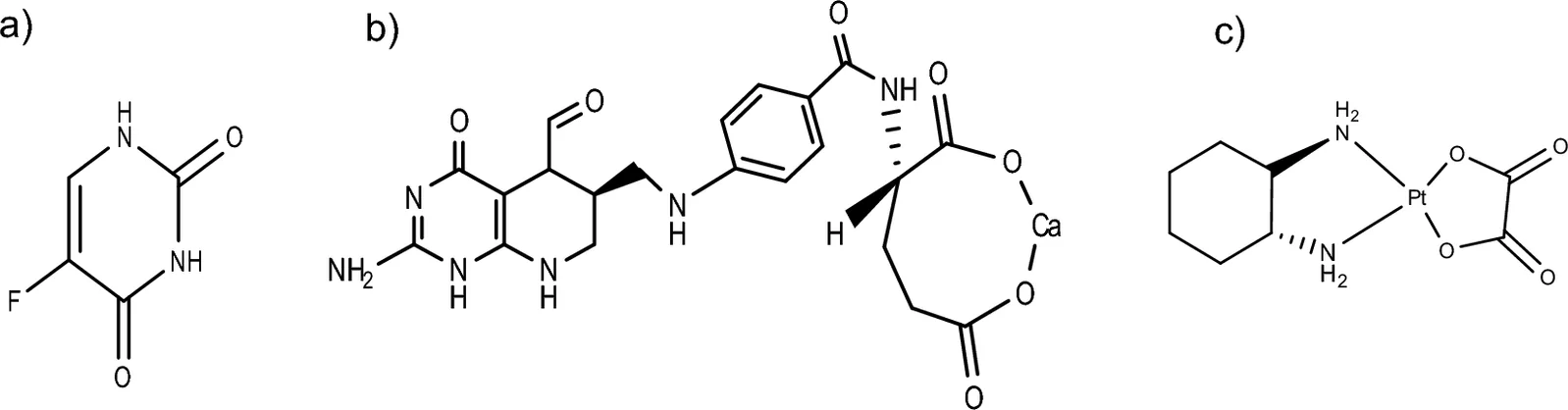
Chemical structures of the studied compounds, (**a**) 5-Fluorouracil (5-FU), (**b**) Leucovorin (LU), and (**c**) Oxaliplatin (OXA).

Colon cancer (CC) represents one of the most significant global health challenges, ranking among the most frequently diagnosed malignancies and the leading causes of cancer-related mortality worldwide^1^. Among the established therapeutic strategies, the FOLFOX regimen, consisting of 5-fluorouracil (5-FU), oxaliplatin (OXA), and leucovorin (LU), remains a cornerstone treatment for stage III colon cancer^2–4^. Within this triad, 5-FU interferes with DNA/RNA synthesis^5^, LU enhances 5-FU cytotoxicity via thymidylate synthase stabilization^6^. OXA induces ribosome biogenesis stress and DNA cross-linking^7^. Owing to the complementary mechanisms of action of its components, FOLFOX has demonstrated substantial improvements in clinical response, progression-free survival (PFS), and overall survival (OS)^8,9^. Given the clinical importance of these agents and their sequential administration in routine practice, the development of reliable analytical methods for their simultaneous determination remains highly desirable^10^. Chemical structures of 5-FU, LU, and OXA are shown in Fig. 1

From an analytical perspective, simultaneous determination of the FOLFOX components is particularly challenging because of the severe overlap of their UV absorption spectra. Conventional zero-order spectrophotometric methods are unable to achieve adequate selectivity without prior separation. Although chromatographic techniques such as HPLC are considered reference methods, reported methods for the FOLFOX regimen require analysis times ranging from 68 min^11^ to 20 min per sample, the latter was achieved in a recent protocol developed by our research team^12^, they also involve the use of organic mobile phases. These characteristics have encouraged the development of alternative approaches capable of reducing analytical complexity while maintaining acceptable analytical performance. Consequently, there is a growing interest in developing sustainable alternatives capable of replacing physical separation with mathematical signal resolution^13^.

Recent advances in chemometric-assisted analysis move away from resource-intensive chromatographic methods toward data-driven resolution strategies for resolving highly overlapping spectral systems. Multivariate calibration approaches such as principal component regression (PCR) and partial least squares (PLS) have demonstrated excellent resolving capabilities; however, their implementation generally requires training datasets, calibration model construction, model optimization, validation procedures, and dedicated chemometric software packages such as MATLAB® or Unscrambler®. In addition, model performance is strongly influenced by the quality and representativeness of the calibration dataset^14^. In contrast, Fourier function convolution enables mathematical resolution of highly overlapped signals, improving selectivity and quantitative performance with no need of model construction, and has been successfully applied to resolve various types of analytical signals^15–18^.

More specifically, the hybrid double divisor ratio spectra (HDDR) approach achieves direct mathematical resolution of overlapping spectra without the need for calibration matrices, training datasets, or multivariate model construction. In addition, HDDR-based spectrophotometry eliminates chromatographic separation and the associated consumption of mobile phases, thereby simplifying the analytical workflow and increasing sample throughput. These features reduce analysis time, reagent consumption, and operational cost while maintaining satisfactory analytical performance. Therefore, HDDR represents a practical alternative that can be readily implemented in routine quality-control laboratories using conventional spreadsheet software^19^.

To the best of our knowledge, this study represents the first application of the HDDR approach for the simultaneous determination of the complete FOLFOX regimen (OXA, 5-FU, and LU) based on simply spectrophotometric measurements. While HDDR has previously been reported as a general strategy for resolving ternary mixtures^19^, it has not been applied to this clinically important and analytically challenging combination. Furthermore, unlike previously reported Fourier-based ratio spectrophotometric methods, HDDR combines the selectivity of the double-divisor concept with Fourier convolution to achieve complete resolution of the severely overlapping spectra of the three drugs while minimizing noise amplification commonly associated with derivative processing. Although multivariate calibration methods may offer advantages for highly complex datasets, HDDR was selected in the present work because it provides direct spectral resolution, operational simplicity, enhanced accessibility, and satisfactory analytical performance without the need for sophisticated chemometric modeling.

The proposed method was validated according to ICH guidelines^20^ and a comprehensive sustainability, practicality, and innovation assessment framework including AES, AGREE, MoGAPI, AGSA, CaFRI, BAGI, RGB, and VIGI metrics, providing a sustainable and practical alternative for pharmaceutical quality control.

## Theoretical background

Primary challenge in spectrophotometric multi-component analysis is the simultaneous determination of two or more active compounds in the same mixture without preliminary separation. Several spectrophotometric methods have been established for resolving mixtures with overlapping spectra, including classical derivative spectrophotometry^21,22^, Vierordt's method^23^, orthogonal function method^24^, Fourier functions method^25,26^, least square method^27^, principal component regression (PCR)^28,29^, and multi-wavelength linear regression analysis (MLRA)^30^. However, when absorbance data is subject to severe spectral overlaps, standard treatments often fail to provide sufficient resolution, necessitating more specialized data processing techniques. ​Consequently, the treatment of absorbance ratio spectra has become a fundamental basis for analytical procedures designed to generate signals depending only on a single analyte. Early developments included the ratio-derivative spectrophotometry proposed by Salinas et al. for binary mixtures^31^, and the derivative ratio spectra-zero-crossing (DRSZ) technique introduced by Berzas Nevado et al. for ternary systems^32^. This evolution led to the “double divisor ratio spectra derivative method” (DDRD), which utilizes a binary sum of two spectra as a divisor to isolate the target analyte's signal^28^. While these derivative-based approaches are effective, they are inherently limited by the degradation in the signal-to-noise ratio that inevitably accompanies the derivative process. To overcome these limitations, the hybrid double divisor ratio spectra (HDDR) method was introduced by integrating the double divisor concept with trigonometric Fourier function processing. This approach enables selective extraction of analyte-specific signals while improving the signal-to-noise ratio. The method relies on mathematical processing of the ratio spectra obtained using an optimized double divisor, leading to effective digital separation of highly overlapped ternary mixtures and providing a reliable alternative to derivative-based procedures^19^. ​

### Mathematical sequence of the hybrid double divisor ratio spectra method (HDDR)^19^ ​

If a ternary mixture of three analytes, *X*, *Y* and *Z*, is considered and if the absorbance value of this ternary mixture is measured at λ*i*, the following equation can be written as

1 $${{\boldsymbol{A}}}_{\mathrm{m},\uplambda \mathrm{i}}={{\boldsymbol{\alpha}}}_{\left\{{\boldsymbol{X}},{\boldsymbol{\lambda}}\right\}}{{\boldsymbol{C}}}_{{\boldsymbol{X}}}+{{\boldsymbol{\beta}}}_{\left\{{\boldsymbol{Y}},{\boldsymbol{\lambda}}\right\}}{{\boldsymbol{C}}}_{{\boldsymbol{Y}}}+{{\boldsymbol{\gamma}}}_{\left\{{\boldsymbol{Z}},{\boldsymbol{\lambda}}\right\}}{{\boldsymbol{C}}}_{{\boldsymbol{Z}}}$$

where A_m,λi_ is the absorbance of the mixture at wavelength λ*i* and ${{\boldsymbol{\alpha}}}_{\left\{{\boldsymbol{X}},{\boldsymbol{\lambda}}\right\},}{{\boldsymbol{\beta}}}_{\left\{{\boldsymbol{Y}},{\boldsymbol{\lambda}}\right\}}$ $\mathrm{a}\mathrm{n}\mathrm{d}{{\boldsymbol{\gamma}}}_{\left\{{\boldsymbol{Z}},{\boldsymbol{\lambda}}\right\}}$ are the absorptivities of *X,Y* and Z, respectively. ${{\boldsymbol{C}}}_{{\boldsymbol{X}}}$, ${{\boldsymbol{C}}}_{{\boldsymbol{Y}}}$ and ${{\boldsymbol{C}}}_{{\boldsymbol{Z}}}$ represent the concentrations of compounds.

The absorbance spectrum of the ternary mixture is divided by a suitable double divisor composed of the spectra of the interfering components. This transformation eliminates the constant contribution and isolates the analytical information of the target compound.

2 $$\frac{{{\boldsymbol{A}}}_{\left\{{\boldsymbol{m}},{\boldsymbol{\lambda}}\right\}}}{{{\boldsymbol{\alpha}}}_{\left\{{\boldsymbol{X}},{\boldsymbol{\lambda}}\right\}}{{\boldsymbol{C}}}_{{\boldsymbol{X}}}^{0}+{{\boldsymbol{\beta}}}_{\left\{{\boldsymbol{Y}},{\boldsymbol{\lambda}}\right\}}{{\boldsymbol{C}}}_{{\boldsymbol{Y}}}^{0}}={\boldsymbol{K}}\left({\boldsymbol{C}}{\boldsymbol{o}}{\boldsymbol{n}}{\boldsymbol{s}}{\boldsymbol{t}}{\boldsymbol{a}}{\boldsymbol{n}}{\boldsymbol{t}}\right)+\frac{{{\boldsymbol{\gamma}}}_{\left\{{\boldsymbol{Z}},{\boldsymbol{\lambda}}\right\}}{{\boldsymbol{C}}}_{{\boldsymbol{Z}}}}{{{\boldsymbol{\alpha}}}_{\left\{{\boldsymbol{X}},{\boldsymbol{\lambda}}\right\}}{{\boldsymbol{C}}}_{{\boldsymbol{X}}}^{0}+{{\boldsymbol{\beta}}}_{\left\{{\boldsymbol{Y}},{\boldsymbol{\lambda}}\right\}}{{\boldsymbol{C}}}_{{\boldsymbol{Y}}}^{0}}$$

According to the basic principle for the use of the trigonometric Fourier functions to process absorbance ratio spectra, an absorbance ratio spectrum can be expanded in terms of the Fourier series as follows^19^:

3 $${{\boldsymbol{A}}}_{{\boldsymbol{r}}\left({\boldsymbol{\lambda}}\right)}={{\boldsymbol{a}}}_{0}+{{\boldsymbol{a}}}_{1}\mathbf{cos}{\boldsymbol{x}}+{{\boldsymbol{a}}}_{2}\mathbf{cos}2{\boldsymbol{x}}+{{\boldsymbol{a}}}_{3}\mathbf{cos}3{\boldsymbol{x}}+\dots +{{\boldsymbol{b}}}_{1}\mathbf{sin}{\boldsymbol{x}}+{{\boldsymbol{b}}}_{2}\mathbf{sin}2{\boldsymbol{x}}+{{\boldsymbol{b}}}_{3}\mathbf{sin}3{\boldsymbol{x}}+\dots $$

where A_r_(λ) denotes the absorbance ratio, obtained by using a double divisor, at a set of (n + 1) equally spaced wavelengths, a_j_ are the coefficients of cos and bj are the coefficients of sin *jx* (= 1, 2, 3, …, n). *a*0 is the constant component, cos ${\boldsymbol{x}}$ is the quadratic component, etc. of the absorbance ratio gross curve.

The optimum coefficient corresponds to a maximum or a minimum in the transformed double divisor ratio spectrum. Such spectrum is obtained by plotting the ${t^{\prime}}_{rj}$ coefficients versus *λ m* (the mean of the wavelengths set). The optimized coefficient ${t^{\prime}}_{rj}$ is proportional to the concentration of the analyte.

4 $${{\boldsymbol{t^{\prime}}}}_{{\boldsymbol{r}}{\boldsymbol{j}}}={\boldsymbol{\alpha}}{\boldsymbol{C}}$$

where *α * is a constant and *C* is the concentration of the analyte. In the case of multi-component mixtures, e.g. a ternary mixture of three components *X, Y* and *Z*, the absorbance spectra of the mixtures are divided by sum of the spectra of *X* and *Y* as a double divisor, the obtained ratio spectra are convoluted using the selected combined trigonometric Fourier functions and the observed coefficients are then plotted against λ_m_. The complete mathematical derivation, including intermediate equations and coefficient calculations, is provided in Supplementary Information (section S1).

## Experimental

### Instrumentation

Spectrophotometric measurements were performed using a Shimadzu UV-1800 double-beam UV–Vis spectrophotometer (Shimadzu Corporation, Kyoto, Japan) with 1.0-cm matched quartz cuvettes. Spectral data acquisition was operated via UVProbe software (Version 2.42, Shimadzu Corporation, Kyoto, Japan). The absorbance data were recorded between 200 and 460 nm at a 2 nm interval using a bandwidth of 2 nm and a scan speed of 600 nm/min. Adjusting pH of solutions was done with a JENWAY Model 3505 pH meter (Jenway, Staffs, UK). All mathematical processing, Fourier-based chemometric transformations, and data analysis were conducted using Microsoft Excel® 365 (Microsoft Corporation, Redmond, Washington, USA).

### Materials and reagents

5-Fluorouracil (5-FU) (Utoral®, 500 mg/10mL), Oxaliplatin (OXA) (Oxaliplatin ®,100 mg/20mL), and Leucovorin (LU) (Calcifolinon®, 100 mg/10mL) were obtained from the local market, with specific lot numbers 220145, 220,038 (Hikma Specialized Pharmaceuticals, Cairo, Egypt), and 23,117,001 (Global Pharmaceutical Industries, 6th October City, Egypt), respectively. Analytical grade sodium hydroxide (NaOH, 98.0% purity) and hydrochloric acid (HCl, 37%v/v, analytical grade) were provided by El-Nasr Chemical Industry Company in Egypt, while HPLC-grade methanol was supplied from Sigma-Aldrich Chemie GmbH, Switzer-Land Germany. High-purity deionized water filtered through a 0.4 µm membrane filter (Sartorius AG, Göttingen, Germany) was used for all solution preparations.

### General procedure and construction of calibration graphs

#### Stock solutions

Primary stock solutions of OXA, 5-FU, and LU were prepared in deionized water to obtain a concentration of 1000 µg/mL by transferring the calculated aliquots of drugs into volumetric flasks and making up to the mark with deionized water. To prevent degradation and ensure stability, these stock solutions were freshly prepared daily and were used immediately to prepare the subsequent working standards.

#### Working standard solutions

To prepare the calibration sets, different aliquots of the prepared stock solutions (1000 µg/mL) were transferred into separate series of 10-mL volumetric flasks. The concentrations were adjusted to ranges of 5–100, 5–75, and 5–50 µg/mL for OXA, 5-FU, and LU, respectively, and the solutions were completed to the mark with deionized water.

#### Preparation of laboratory prepared mixtures

Laboratory prepared mixtures with different concentration ratios of the studied drugs were prepared in deionized water within the linearity range as follows: (8 µg/mL OXA, 6 µg/mL 5-FU, and 10 µg/mL LU), (45 µg/mL OXA, 30 µg/mL 5-FU, and 24 µg/mL LU), (90 µg/mL OXA, 60 µg/mL 5-FU, and 45 µg/mL LU), (50 µg/mL OXA, 50 µg/mL 5-FU, and 10 µ g/mL LU), (50 µg/mL OXA, 50 µg/mL 5-FU, and 50 µg/mL LU), (10 µg/mL OXA, 50 µg/mL 5-FU, and 50 µg/mL LU), (15 µg/mL OXA, 75 µg/mL 5-FU, and 50 µg/mL LU). It is important to mention that the final two mixtures were specifically prepared to reflect the therapeutic dose ratios administered during the first phase of the clinical protocol. These ratios correspond to the concomitant administration of OXA (85–100 mg/m^2^) and LU (400 mg/m^2^) over 120 min, followed by the 5-FU bolus (400 mg/m^2^)^10^. This ensures that the analytical method is validated against drug proportions that strictly follow the established FOLFOX dosage regimen.

## Application of analytical methods and construction of calibration graphs

### Direct spectrophotometric method

**Fig. 2:**
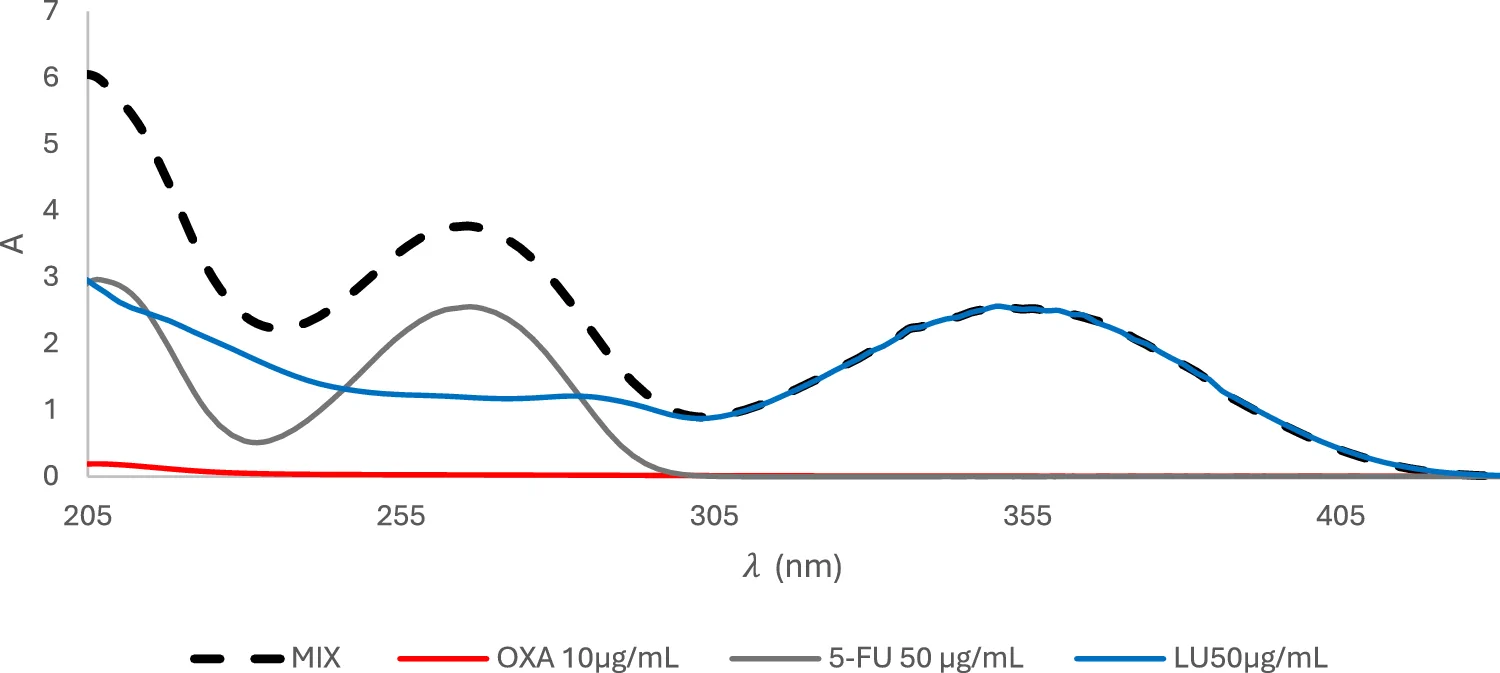
UV absorption spectra of standard solutions of 10 µg/ mL OXA, 50 µg /mL 5-FU and 50 µg /mL LU and their mixture in deionized water

Zero-order spectra (D0) of OXA, 5-FU, and LU standard solutions, as well as their synthetic mixtures were scanned within the wavelength range of 200–460 nm at intervals of 2 nm. All measurements were performed against deionized water as blank. OXA and 5-FU showed complex spectral overlap between 200 and 300 nm while LU displayed a clear distinct peak at 355 nm. Thus, the absorbance values of LU standard solutions at 355 nm were measured and plotted against their corresponding concentrations to create the calibration graph. LU concentrations in its synthetic mixtures were determined using the obtained regression equation (Figs.2 and S1).

### First-derivative spectrophotometric method

**Fig. 3:**
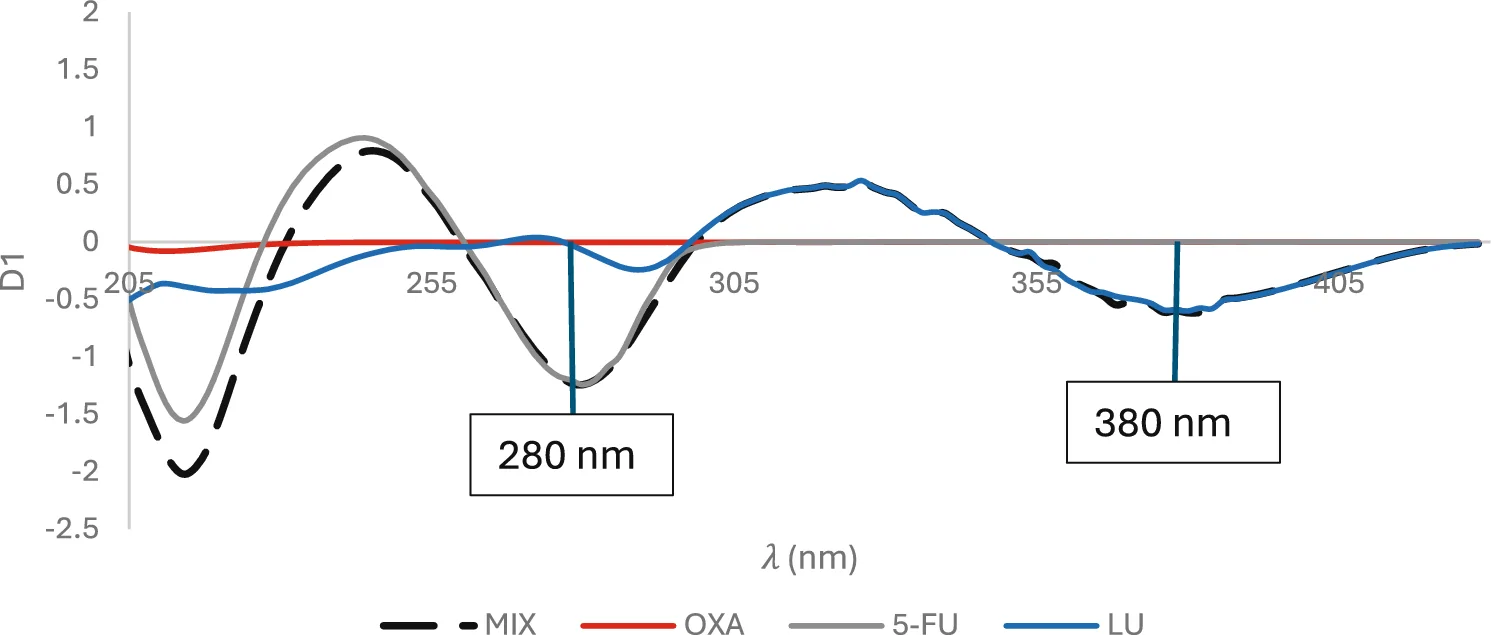
First derivative spectra of standard solutions of 10 µg/ mL OXA, 50 µg /mL 5-FU, and 50 µg /mL LU and their mixture in deionized water (∆λ =10 nm)

**Fig. 4:**
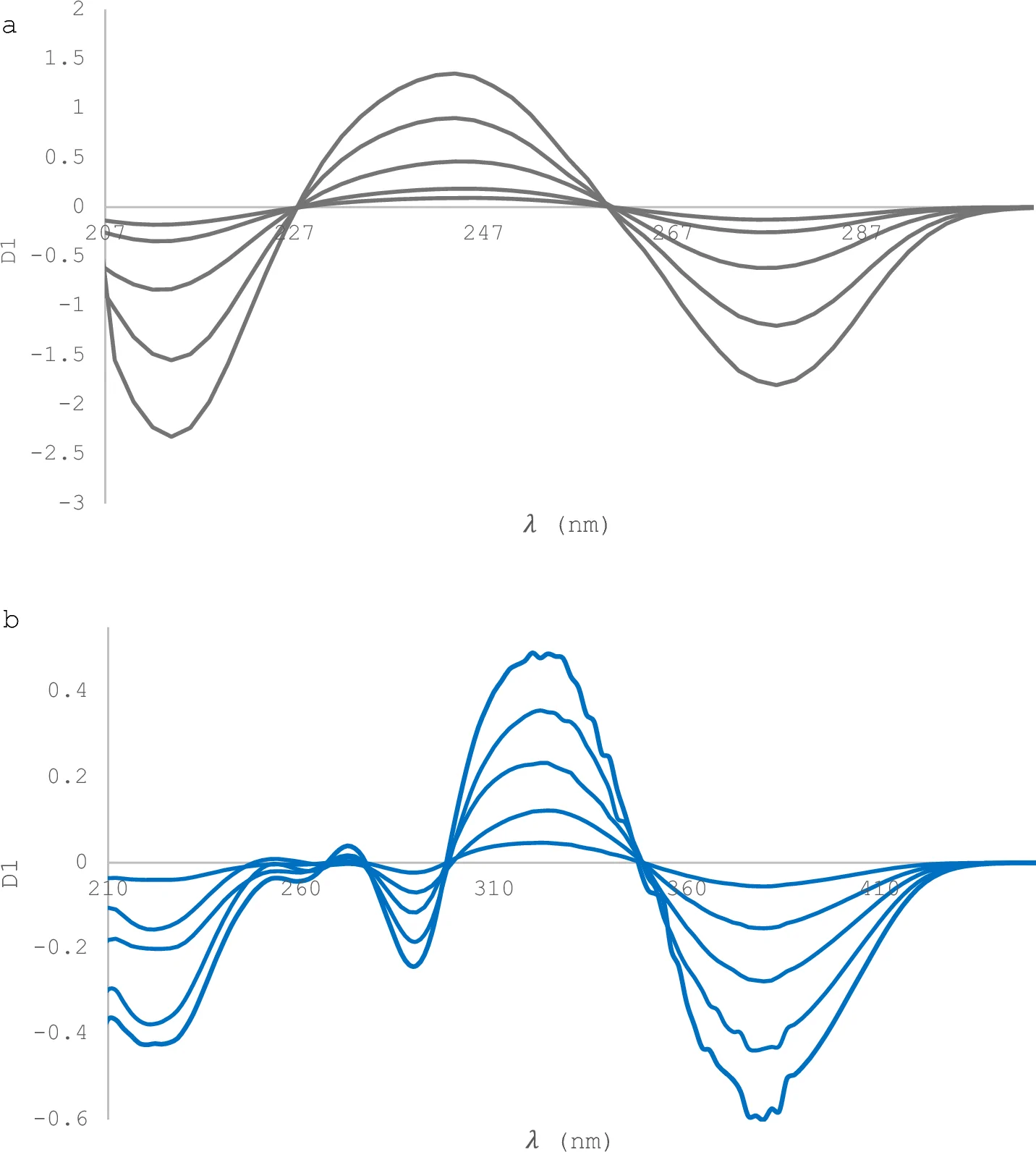
(**a**) First derivative spectra of standard solutions of 5-FU in deionized water at the linearity range (5–75 µg/mL) showing the selected λ to solve 5-FU (280 nm), (**b**) First derivative spectra of standard solutions of LU in deionized water at the linearity range (5–50 µg/mL) showing the selected λ to solve LU (380 nm).

First derivative spectra (D1) of the above prepared ternary mixtures (OXA, 5-FU, and LU) (Fig. 3 and S2) along with their individual drugs standard solutions (Fig. 4a and b) were recorded in deionized water against blank with a scaling factor (∆λ= 10 nm). This method was applied to allow the determination of 5-FU and LU based on the zero-crossing technique. For 5-FU, the D1 spectrum of the mixture exhibited a distinct peak at 280 nm, where zero-crossing points of OXA and LU were detected. For LU, another measurable peak was identified at 380 nm. At this wavelength, both OXA and 5-FU reached their zero-crossing points. Thus, the derivative amplitude at 380 nm was directly proportional to the concentration of LU. Finally, the obtained D1 amplitudes at 280 nm for 5-FU and 380 nm for LU were plotted against their corresponding concentrations and their regression equations were computed.

### Hybrid double divisor ratio spectra method (HDDR)

​This method was applied to resolve severe spectral overlaps in the ternary mixture by convoluting the ratio spectra using combined trigonometric Fourier functions cos *xi *polynomials. The procedure started with recording the absorption spectra of OXA, 5-FU, and LU standard solutions alongside their ternary mixtures, each spectrum was divided by a double divisor which was mathematically derived from the sum of the absorption spectra of the other two components at specific concentrations. ​The Fourier function coefficients (*t*′_*r*_) were calculated for each drug using eight equally spaced wavelengths at 2 nm intervals, according to the following equation.

5 $${{\boldsymbol{t}}}_{{\boldsymbol{r}}{\boldsymbol{j}}}^{\boldsymbol{^{\prime}}}=\frac{\left[\begin{array}{c}\left(+1.707\right){A}_{r0}+ \left(+0.707\right){A}_{r1} + \left(-0.707\right){A}_{r2} + \left(-1.707\right){A}_{r3} +\\ \left(-1.707\right){A}_{r4} + \left(-0.707\right){A}_{r5}+ \left(+0.707\right){A}_{r6} + \left(+1.707\right){A}_{r7}\end{array}\right]}{4}$$

where ${A}_{r0}$–${A}_{r7}$ stand for the eight absorbance ratio values. The coefficients were derived from the combined Fourier function $T^{\prime} = [cos x + cos(x + 45)].$ ​ To establish the calibration graphs, the *t*′_*r*_ values were measured from the convoluted ratio spectra of pure standard solutions as follows:

For the determination of OXA.

**Fig. 5:**
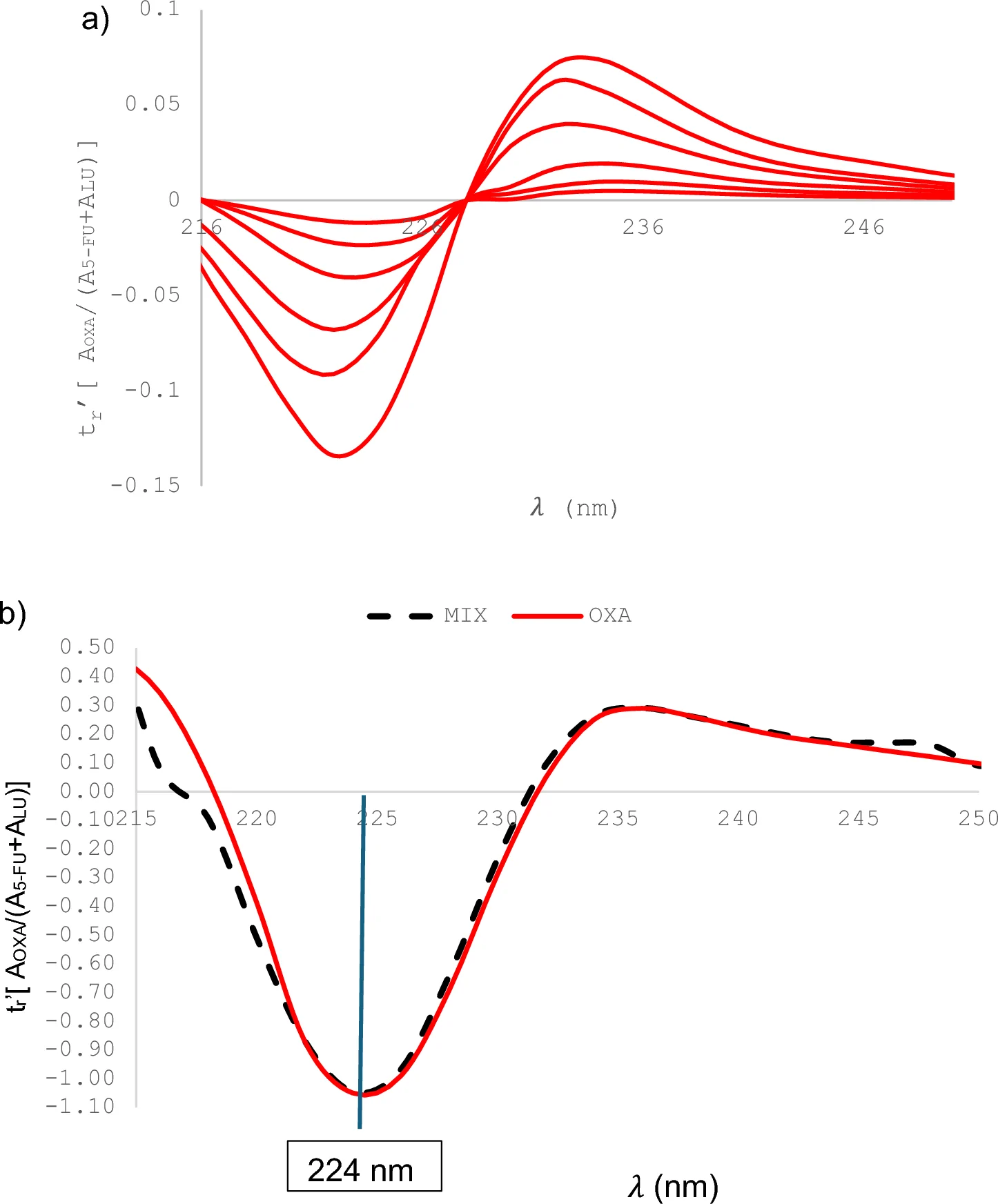
(**a**) Convoluted ratio spectra using eight-point combined trigonometric Fourier functions of OXA (5, 10, 25, 50, 80 and 100 µg/mL) and (**b**) The coincident spectra of the convoluted ratio spectra using combined trigonometric Fourier function of 10 µg/mL OXA and ternary mixture (10 µg/ mL OXA, 50 µg /mL 5-FU, and 50 µg /mL LU) using (25 µg/mL 5-FU + 5 µg/mL LU) as double divisor in deionized water.

The stored spectra of serial dilutions of OXA standards were divided by a sum of the spectra of 25 µg/mL 5-FU and 5 µg/mL LU, after that the *t*′_*r*_ amplitudes were recorded at 224 nm (Fig. 5a). ​

**Fig. 6:**
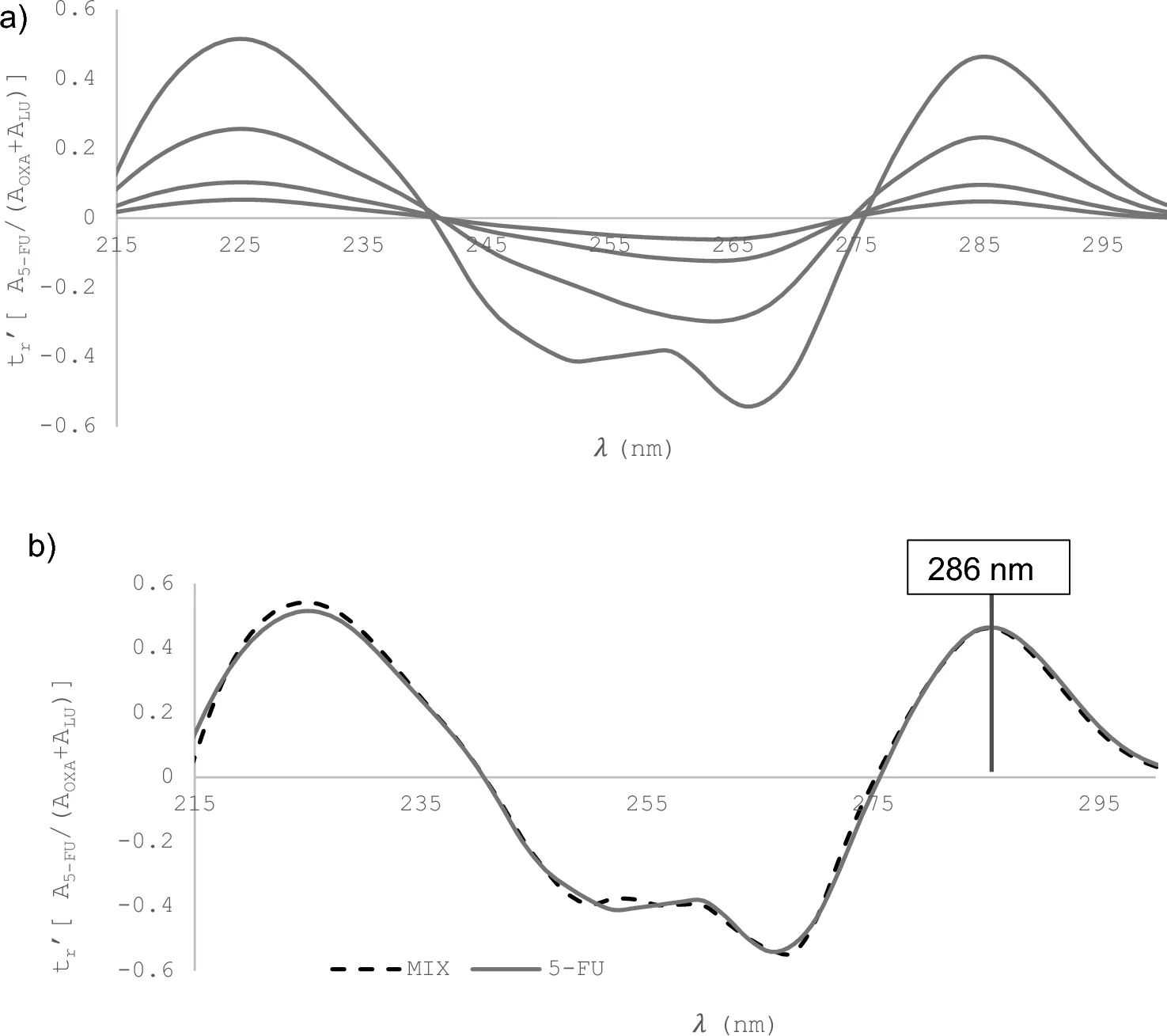
(**a**) Convoluted ratio spectra using eight-point combined trigonometric Fourier functions of 5-FU (5, 10, 25, 50, 75 µg/mL) and (**b**) The coincident spectra of the convoluted ratio spectra using combined trigonometric Fourier function of 50 µg/mL 5-FU and ternary mixture (50 µg/ mL OXA, 50 µg /mL 5-FU, and 10 µg /mL LU) using (10 µg/mL OXA + 10 µg/mL LU)as double divisor in deionized water.

**Fig. 7:**
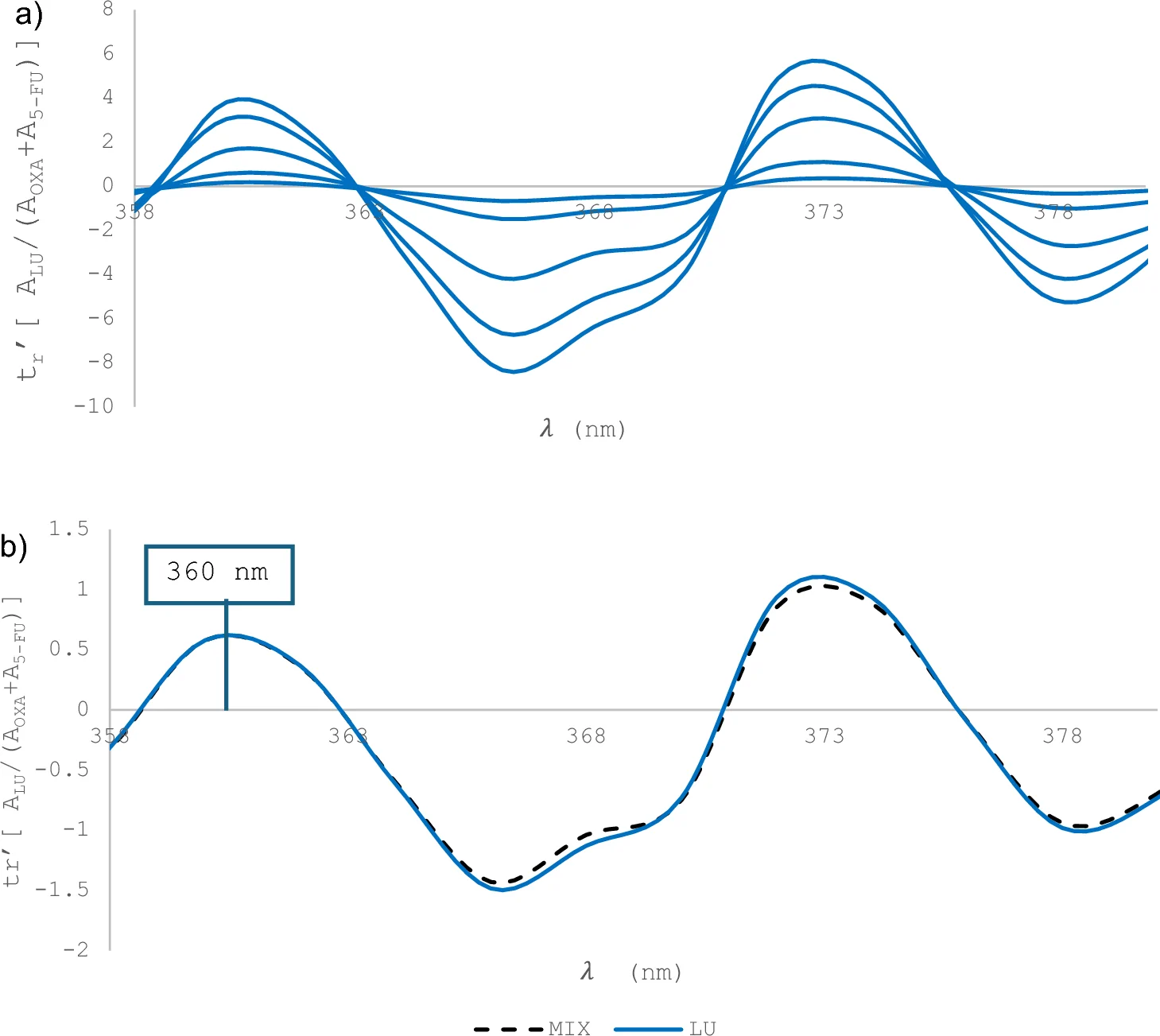
(**a**) Convoluted ratio spectra using eight-point combined trigonometric Fourier functions of LU (5, 10, 25, 40, 50 µg/mL) and (**b**) The coincident spectra of the convoluted ratio spectra using combined trigonometric Fourier function of 10 µg/mL LU and ternary mixture (50 µg/ mL OXA, 50 µg /mL 5-FU, and 10 µg /mL LU) using (25 µg/mL OXA + 5 µg/mL 5-FU) as double divisor in deionized water.

For the determination of 5-FU, 5-FU standard solutions spectra reflecting the linearity range were divided by the sum of spectra of 10 µg/mL OXA and 10 µg/mL LU as a double divisor, then the *t*′_*r*_ values were obtained at 286 nm (Fig. 6a). In the same way for the determination of LU, LU standards at subsequent concentrations were divided by the sum of spectra of 25 µg/mL OXA and 5 µg/mL 5-FU and, the *t*′_*rj*_ values were collected at 360 nm (Fig. 7a).

​The resulting *t*′_r_ amplitudes of the three drugs were plotted versus the respective concentrations of each drug to obtain the regression equations.

## Results and discussion

The analytical capability of the proposed HDDR method is demonstrated by successfully isolating the three components OXA, 5-FU, and LU. The three cited medications compose the FOLFOX chemotherapy, which is the standard protocol used to treat colorectal cancer. Their clinical administration involves the co-infusion of OXA and LU via Y-site infusion followed by 5-FU bolus injection, thus it highlights the need for a reliable method for their simultaneous determination. The proposed method depends on the spectrophotometric determination of the cited drugs since the three studied drugs exhibit extensive spectral overlap in the UV region (Fig. 2). This complexity prevents direct univariate spectrophotometry from their simultaneous determination without prior separation only LU showed distinct absorbance and could be directly determined by direct absorbance measurement. Derivative treatment of absorbance spectra could only determine 5-FU and LU. Accordingly, the proposed HDDR was unique in its ability to resolve the spectral complication of the ternary mixture with successful determination of each of the three cited drugs.

​ Literature review revealed two published reports based on HPLC methods for the stability-indicating studies of these drugs in different matrices, these approaches had a major drawback of prolonged run times (20 to 68 min)^11,12^. In contrast, the proposed HDDR method provides a fast and efficient mathematical resolution of the overlapping bands. This approach is further justified by the drugs' different absorbance coefficients and their negligible chemical interaction, as a criterion necessary for their absorbances to be additive^19^. Consequently, the proposed method effectively resolves these overlapping signals without the requirement of physical separation or time-consuming chromatographic runs. Along with the general advantages of simplicity, availability and cost-effectiveness of spectrophotometry.

### Selection of diluting solvent

To optimize the spectrophotometric determination of 5-FU, OXA, and LU, various solvents including deionized water, 0.1 M NaOH, and 0.1M HCl were evaluated. The selection was primarily governed by the solubility of the analytes and, more critically, their chemical stability within the medium. Although 5-FU showed high solubility in 0.1M NaOH, the alkaline medium was found to be unsuitable for 5-FU itself and OXA, as they undergo hydrolysis, leading to the degradation and subsequent spectral distortion^33,34^. Similarly, the use of 0.1 M HCl was avoided to prevent potential ligand exchange reactions in OXA^35,36^ and the risk of LU hydrolysis^37^. Methanol was excluded due to its lower solubility for OXA^38^ and the presence of significant spectral overlaps that hindered clear resolution. Consequently, deionized water was selected as the optimal solvent; it provided a stable and inert environment that preserved the chemical integrity of all three drugs. It ensured a consistent pH, which is essential for maintaining stable spectral profiles. Furthermore, the use of deionized water aligns with green analytical chemistry principles by avoiding organic solvents and harsh reagents.

### Direct spectrophotometric method

The zero-order absorption spectra (D0) of OXA, 5-FU, and LU showed a high degree of complexity. A visual examination of the spectra showed that OXA and 5-FU had a challenging spectral overlap making it difficult to determine them individually through direct methods without prior separation, but the spectrum of LU had a well-resolved, distinct peak at 355 nm (Figs.2 and S1). At this selective wavelength, OXA and 5-FU didn't exhibit absorbance, allowing for the accurate and direct quantification of LU through its D0 values. Thus, more derivative-based and ratio-processing spectrophotometric methods should be examined to overcome the obstacle of the overlapping bands of OXA and 5-FU.

### First-derivative spectrophotometric method

To resolve the severe spectral overlap, D1 (dA/d λ) signals were measured by utilizing Excel software to differentiate the zero-order absorbance data with respect to wavelength (λ). These derivative signals exhibited clear maxima or minima, providing the simultaneous determination of the studied drugs via the zero-crossing technique.

For 5-Fluorouracil (5-FU), as shown in Figs. 3 and S2, the D1 spectra of the ternary mixture showed a well-defined peak at 280 nm matching a peak that appeared in the D1 spectra of pure 5-FU. At this specific wavelength, both OXA and LU exhibited zero-crossing points, ensuring that the obtained amplitude was proportional to the concentration of 5-FU without any mutual interference.

For leucovorin (LU), a peak recorded at 380 nm was used for its determination since both OXA and 5-FU showed zero-crossing points (Fig. 3). This provided a selective and independent approach for LU determination. Although the D1 method showed selective peaks for the determination of both 5-FU and LU, direct determination of OXA was not achievable in its ternary mixtures, since no single wavelength could be selected with zero response of both of the other two drugs, 5-FU and LU.

To resolve the interference hindering the direct determination of OXA, higher-order derivative spectra D2, D3, and D4 were further explored. However, increasing the derivative order did not offer a significant advantage; the signals remained severely overlapped, and no specific wavelength could be identified where both 5-FU and LU simultaneously reached a zero-crossing point, making the quantitative measurement of OXA unreliable in the ternary mixture using derivative spectrophotometric method.

### Optimization of experimental conditions

​ The effect of the wavelength interval (∆λ) was investigated to achieve the highest sensitive D1 signals with the least amount of noise. When different wavelength intervals were examined, it was found that ∆λ = 10 nm provided the ideal balance of peak sharpness with effective wise smoothing baseline. Smaller intervals resulted in noisy signals, whereas larger intervals led to significant peak broadening and loss of important spectral details. In addition, medium scanning speed was chosen to ensure the best of absorbance characteristics of the derivative amplitudes, facilitating the precise and selective resolution of 5-FU and LU.

### Hybrid double divisor ratio spectra method (HDDR)

Total resolution of the ternary mixture of OXA, 5-FU, and LU is considered an analytical challenge because of their complicated spectral overlaps. In this respect, HDDR method was developed and was used as a more powerful and precise option. This hybrid method combines the selectivity of the “Double Divisor” technique with the mathematical accuracy of Fourier convolution, making it a powerful tool for separating the signals of interest from their heavily overlapped spectra^19^. ​

For the quantification of OXA, the most challenging analyte in the mixture, the combined absorption spectra of 5-FU and LU (25 and 5 µg/mL, respectively) were utilized as a double divisor. This step was essential to extract the OXA signal from the mutual interferences of the other two drugs. The resulting ratio spectra were then subjected to a convolution process using an eight-point combined trigonometric Fourier function, *T*′ = [*cosx* + *cos*(*x* + 45)], at optimized 2 nm wavelength intervals. By plotting the calculated coefficients (*t*′_*r*_) against the mean wavelength (λ_*m*_), a clear and pure analytical signal for OXA was successfully resolved at λ*m* 224.0 nm, measured via the peak-to-zero technique (Fig. 5a).

In the same way, 5-FU could be quantified by using a double divisor of OXA and LU (10 µg/mL each). Convolution of HDDR spectra enabled the determination of 5-FU at its own independent identity at λ_*m*_ 286.0 nm (peak-to-zero), free from any background interference (Fig. 6a). Finally, after applying this method on LU, the HDDR method proved its versatility through accurately capturing the LU signal at λ_*m*_ 360.0 nm (peak-to-zero) with high accuracy and precision (Fig. 7a). ​The fact that the convoluted ratio spectra of the pure drugs perfectly matched those of the ternary mixture at the selected working wavelengths was definitive proof of the method's success which ensures that the Fourier convolution worked well as a digital filter, getting rid of baseline fluctuations and background noise (Fig. 5b,6b, and 7b). The proposed HDDR method successfully achieved the reliable, simultaneous determination of OXA, 5-FU, and LU without the need for any complicated extraction or pre-separation steps at a satisfactory level of resolution and purity.

### Optimization of experimental conditions

Many trials were required to optimize this method owing to the severe spectral overlap between the components OXA, 5-FU and LU. Different Fourier functions, number of points, and wavelength intervals were examined to determine the conditions that produced the most reliable and interference-free separation. It was found that using an eight-point, *T*′ = [*cosx* + *cos*(*x* + 45)], combined trigonometric Fourier functions for convoluting the double divisor absorbance ratio spectra at 2 nm intervals provided the best match of three spectral characteristics.

An especially important parameter to be optimized was the double divisor concentrations. In this respect, numerous combinations and concentration ratios were investigated to identify those that most effectively suppressed the contribution of the interfering components, along with a high degree of sensitivity for the cited component. After careful evaluation, the optimal divisors were determined to be 5‑FU/LU (25:5 μg/mL) for OXA, OXA/LU (10:10 μg/mL) for 5‑FU, and OXA/5‑FU (10:50 μg/mL) for LU determination.

## Validation of the proposed methods

In compliance with ICH Q2(R1) guidelines^20^, the proposed methods validation was referred with respect to linearity, limits of detection and quantitation, precision, accuracy and specificity.

### Calibration curves and statistical data

Under the above-described experimental conditions, at the selected wavelengths, linear graphs were obtained by plotting the absorbance values versus concentration for LU (direct spectrophotometric method), first derivative absorbance values versus concentrations for 5-FU and LU (D1 method) and combined trigonometric Fourier function coefficients (HDDR method) versus concentration for OXA, 5-FU and LU.

In all cases, results of the linear least squares regression treatment of analytical data were also provided including the slopes, intercepts, and correlation coefficients (Table 1). High correlation coefficient values (r-values > 0.999) with negligible intercepts indicated high degree of linearity. Standard deviation of the intercept (S_a_), the slope (S_b_), and the residuals (S_y/x_) were also given. The deviation between the calculated and found (measured) y values was estimated from the calculated (S_y/x_). The closer the experimental points to the regression line, the smaller the (S_y/x_) values. An increase in the variance ratio (F values) for equal degrees of freedom indicated an increase in the mean of squares due to regression and decrease in the mean of squares due to residuals. High values for the (r) and (F) were indicative of good regression lines^39^.

**Table 1:** Analytical parameters for determination of OXA, 5-FU and LU ternary mixture using the proposed spectrophotometric methods.

Analyte	OXA	5-FU		LU		
Method	HDDR	D1	HDDR	D0	D1	HDDR
Wavelength (nm)	224	280	286	355	380	360
Linearity Range (µg/mL)	5–100	5–75	5–75	5–50	5–50	5–50
LOD (µg/mL)	1.35	0.27	0.25	0.22	1.51	0.19
LOQ (µg/mL)	4.10	0.83	0.77	0.70	4.58	0.58
Correlation coefficient	0.999	0.9999	0.9999	0.9992	0.9996	0.9994
r	0.998	0.99998	0.99999	0.998	0.9993	0.9989
F	2119.00	244,075.50	308,592.70	2071.42	4612.68	2871.516
Significance F	1.33 × 10^–6^	1.83 × 10^–8^	1.29 × 10^–8^	2.34 × 10^–5^	7.03 × 10^–6^	1.43 × 10^–5^
S_Y/X_	7.90 × 10^–4^	2.71 × 10^–3^	9.70 × 10^–4^	2.13 × 10^–2^	2.00 × 10^–3^	5.83 × 10^–2^
Slope	− 0.0013	− 0.023	0.009	0.25	− 0.012	0.081
S_b_	1.10 × 10^–5^	4.70 × 10^–5^	1.66 × 10^–5^	5.59 × 10^–4^	1.70 × 10^–4^	0.001
Intercept	− 0.006	− 0.007	0.0016	− 0.031	− 0.0011	− 0.25
S_a_	5.33 × 10^–4^	1.91 × 10^–3^	7.00 × 10^–4^	1.74 × 10^–2^	5.50 × 10^–3^	4.71 × 10^–3^

r, correlation coefficient; S_y/x_, standard deviation of residuals; S_a_, standard deviation of intercept; S_b_, standard deviation of slope; F, variance ratio, equals the mean of squares due to regression divided by the mean of squares about regression (due to residuals); LOD, limit of detection; LOQ, limit of quantitation.

### Detection and quantification limits

The formulae were employed to calculate both the limit of detection (LOD) and limit of quantification (LOQ) based on ICH Q2(R1) guidelines^20^. This was referred by multiplying the following formulae: σ/b by 3.3 and 10 for calculating the LOD and LOQ, respectively, while considering that σ is the standard deviation of y‐intercepts of regression line (S_a_) and b is the slope. Low LOD and LOQ values were achieved for each drug based on the above methods, and the results were confirmed by experimentation (Table 1).

### Accuracy

Accuracy was confirmed by the quantification of OXA, 5-FU or LU in synthetic mixtures at three unique concentration levels. The results obtained were used to calculate the mean % recovery of the studied analyte and Er%. Satisfactory recoveries with relative error < 2% indicated the high degree of accuracy of the proposed methods (Tables 2 and 3).

**Table 2:** Intra-day precision and accuracy for determination of OXA, 5-FU and LU using the proposed spectrophotometric methods.

Method	Nominal value (μg /mL)			Mean %recovery ± SD (μg/ mL)			RSD (%)			E_r_(%)		
	OXA	5-FU	LU	OXA	5-FU	LU	OXA	5-FU	LU	OXA	5-FU	LU
Method I D0			10			100.97 ± 0.18			0.18			0.97
			24			100.64 ± 0.34			0.34			0.64
			45			100.52 ± 0.40			0.40			0.52
Method II D1		6	10		100.94 ± 0.08	99.80 ± 0.19		0.08	0.19		0.94	− 0.20
		30	24		99.88 ± 0.11	100.56 ± 0.42		0.11	0.42		− 0.12	0.56
		60	45		100.06 ± 0.34	100.63 ± 0.54		0.34	0.54		0.06	0.63
Method III HDDR	8	6	10	99.90 ± 0.16	100.44. ± 0.06	99.93 ± 0.11	0.16	0.06	0.11	− 0.10	0.44	− 0.07
	45	30	24	100.30 ± 0.32	100.27 ± 0.16	100.28 ± 0.21	0.32	0.16	0.21	0.30	0.27	0.28
	90	60	45	100.89 ± 0.34	99.89 ± 0.50	100.13 ± 0.31	0.34	0.50	0.31	0.89	− 0.11	0.13

Mean % recovery ± SD, Mean ± standard deviation for three determinations; RSD (%), % Relative standard deviation; Er(%), % Relative error.

**Table 3:** Inter-day precision and accuracy for determination of OXA, 5-FU and LU using the proposed spectrophotometric methods.

Method	Nominal value (μg /mL)			Mean %recovery ± SD (μg/ mL)			RSD (%)			E_r_ (%)		
	OXA	5-FU	LU	OXA	5-FU	LU	OXA	5-FU	LU	OXA	5-FU	LU
Method I D0			10			100. 12 ± 0.35			0.35			0.12
			24			101. 45 ± 0.28			0.28			1.45
			45			99.68 ± 0.52			0.52			− 0.32
Method II D^1^		6	10		99.72 ± 0.15	100.45 ± 0.20		0.15	0.20		− 0.28	0.45
		30	24		99.65 ± 0.40	100.28 ± 0.25		0.40	0.25		− 0.35	0.28
		60	45		101.05 ± 0. 35	100.18 ± 0.50		0.35	0.50		1.05	0.18
Method III HDDR	8	6	10	100.62 ± 0.28	99.45 ± 0.10	100.15 ± 0.18	0.28	0.10	0.18	0.63	− 0.55	0.15
	45	30	24	99.92 ± 0.32	100.82 ± 0.22	99.68 ± 0.35	0.32	0.22	0.35	− 0.08	0.82	− 0.32
	90	60	45	99.35 ± 0.40	100.55 ± 0.48	101.15 ± 0. 55	0.40	0.48	0.54	− 0.65	0.55	1.15

Mean %recovery ± SD, Mean ± standard deviation for three determinations ;RSD (%), % Relative standard deviation ; Er(%), % Relative error.

### Precision

Precision was assessed using three concentration levels within the linearity range of each compound: low, medium, and high. The analysis was performed at two levels: thrice on the same day (intra-day) and three successive days (inter-day). The percentage relative standard deviation (RSD%) was calculated and small values less than 2% were obtained showing a high degree of precision of the proposed methods (Tables 2 and 3).

### Robustness

The robustness was evaluated according to ICH Q2(R1) guidelines^20^ by introducing small, deliberate variations in key operational parameters, including wavelength setting) ± 1.0 nm), divisor concentration (± 2%). As summarized in Table S1, the percentage recovery ranged from 99.05% to 100.65% with relative standard deviations (%RSD) remaining strictly below 1.2%. These small deliberate changes did not significantly affect the quantitative determination of the target analytes, demonstrating the high operational stability and robustness of the proposed method for routine testing (Table S1).

### Specificity

Method specificity was investigated by the analysis of synthetic mixtures at various concentration ratios 1:1:1 and 5:5:1 [OXA : 5-FU : LU] and two mixture solutions with ratios 1:5:5 and 1:3:2 [OXA : 5-FU : LU] simulating the actual therapeutic dose within two first hours of dose administration. The mean % recovery and SD values were sufficient to highlight the great specificity of the techniques suggested for OXA, 5-FU or LU simultaneous analysis in the presence of one another, proving the methods' effectiveness even when applied to actual therapeutic dose ratios (Table 4).

**Table 4:** Assay result for determination of OXA, 5-FU, and LU by the proposed spectrophotometric methods in their laboratory prepared mixtures (mixtures c and d represent actual therapeutic dose after 2h from starting administration of FOLFOX protocol)^10^.

Analyte		OXA	5-FU		LU		
Method		Mean %recovery ± SD (μg /mL)					
		HDDR	D^1^	HDDR	D^0^	D^1^	HDDR
Mixture ratio	1:1:1^a^	98.00 ± 0.52	99.90 ± 0.20	99.99 ± 0.15	101.02 ± 0.50	99.38 ± 0.97	100.58 ± 0.78
	5:5:1^b^	99.91 ± 0.90	100.30 ± 0.35	99.89 ± 0.25	100.16 ± 1.00	100.09 ± 0.77	99.78 ± 0.93
	1:3:2^c^	100.58 ± 0.94	99.89 ± 0.19	100.01 ± 0.14	100.50 ± 0.98	98.43 ± 1.00	99.55 ± 0.57
	1:5:5^d^	98.85 ± 0.50	100.09 ± 0.50	101.00 ± 0.82	99.85 ± 0.79	101.80 ± 0.25	101.00 ± 0.44

Ternary mixture ^a^50 µg/ mL OXA, 50 µg /mL 5-FU, and 50 µg /mL LU, ^b^50 µg/ mL OXA, 50 µg /mL 5-FU, and 10 µg /mL LU.

^c^15 µg/ mL OXA, 75 µg /mL 5-FU, and 50 µg /mL LU, ^d^ 10 µg/ mL OXA, 50 µg /mL 5-FU, and 50 µg /mL LU.

### Comparison with the reported methods

A comparison with the reported HPLC methods^11,12^ revealed clear differences in analytical workflow, sample throughput, and resource consumption. The reported chromatographic procedures require analysis times ranging from 20^12^ to more than 68^11^ min per sample, corresponding to sample throughputs of approximately 3 and 0.88 samples h^−1^, respectively. In contrast, the proposed HDDR method relies on direct spectrophotometric measurements combined with mathematical signal resolution, eliminating the need for chromatographic separation and mobile-phase consumption. Consequently, a substantially higher sample throughput can be achieved, making the method particularly attractive for routine quality-control applications. Although HPLC remains a reference analytical technique, the proposed method demonstrated satisfactory analytical performance according to ICH validation criteria^20^. The obtained linearity (r > 0.999), accuracy, precision (%RSD < 2), specificity, and robustness were comparable to those reported for validated HPLC procedures. Furthermore, sustainability assessment demonstrated improved environmental and practical profiles for the proposed method relative to the reported HPLC procedures, as evidenced by the greenness, blueness, and whiteness metrics presented in the following sections (Table S2).

### Greenness evaluation

Environmental sustainability has become an essential criterion in the development and selection of analytical methodologies^40–44^. Therefore, the environmental profiles of the proposed methods and the reported methods^11,12^ were comprehensively assessed using several complementary green metrics, namely AES, AGREE, MoGAPI, AGSA, and CaFRI. Although these tools evaluate different aspects of method greenness, they collectively provide a comprehensive assessment of the environmental impact and overall performance of analytical procedures.

#### Analytical eco-scale (AES)

The analytical eco-scale (AES) evaluates the environmental friendliness of analytical methods based on reagent hazards, energy consumption, occupational risks, and waste generation. Generally, methods with scores above 75 are considered environmentally acceptable^45^. As shown in Tables 5 and S3, The proposed HDDR method achieved an AES score of 96 compared with 84 for the reported HPLC methods^11,12^. This improvement can be attributed primarily to the use of water as the analytical medium and the elimination of solvent-intensive chromatographic separations. Consequently, the proposed method generated lower chemical waste while reducing analyst exposure to hazardous reagents.

**Table 5:**
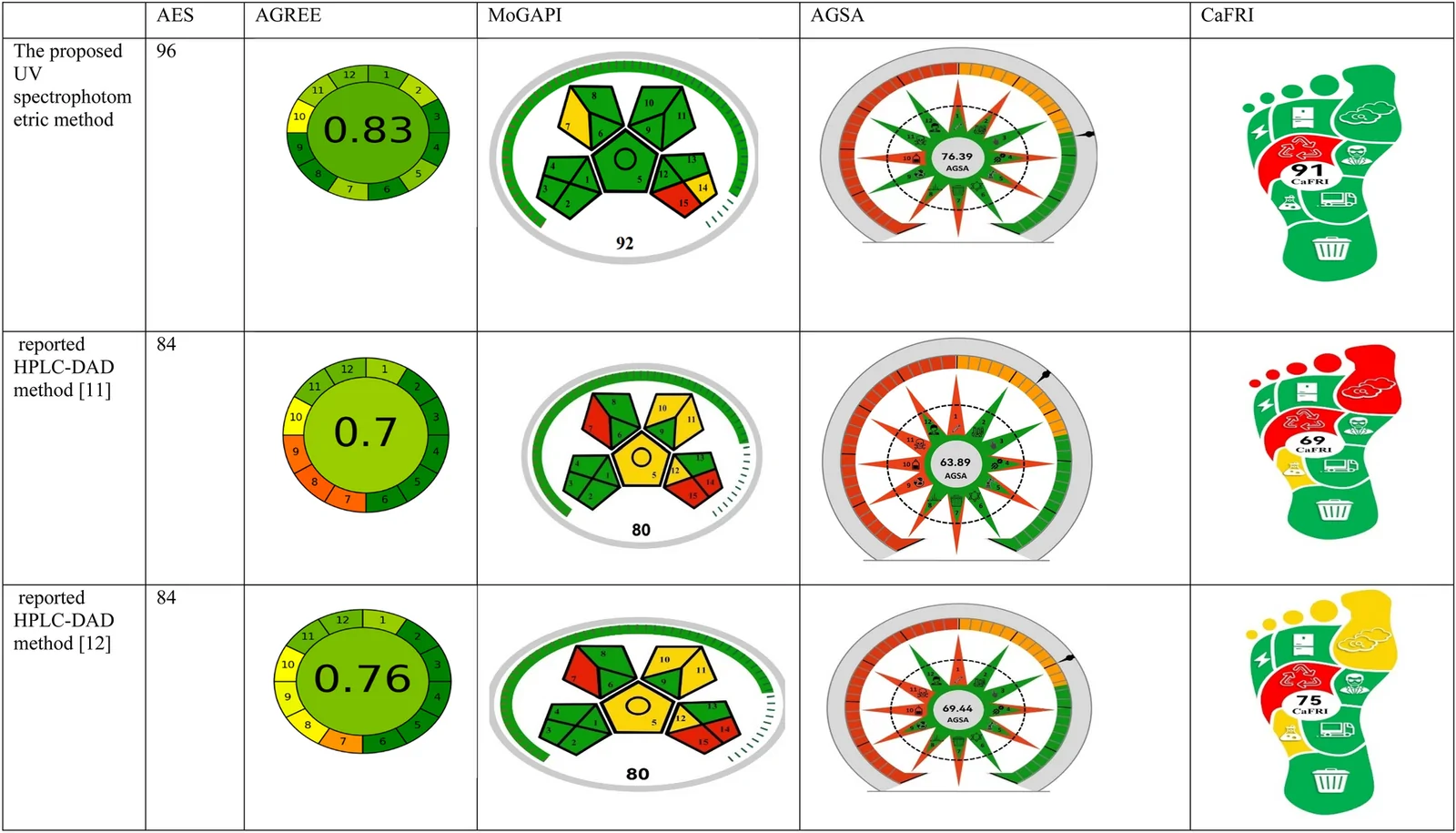
Evaluation of the degree of greenness of the proposed spectrophotometric method in comparison with the reported HPLC–DAD methods^11,12^ using AES, AGREE, MoGAPI, AGSA and CaFRI.

#### Analytical greenness metric (AGREE)

The AGREE metric provides a holistic assessment of compliance with the twelve principles of green analytical chemistry through a unified score ranging from 0 to 1, where higher values indicate better environmental performance^46^. The proposed HDDR method achieved an AGREE score of 0.83. This favorable score mainly reflects reduced waste generation (sector 7), lower energy requirements (sector 9), and the simplified analytical workflow associated with direct spectrophotometric measurement. In comparison with chromatographic methods^11,12^ requiring mobile phases and prolonged runtimes (sector 8), the proposed procedure exhibits improved compliance with several green analytical chemistry principles (Table 5).

#### The modified GAPI (MoGAPI)

The modified green analytical procedure index (MoGAPI) evaluates the environmental impact of the complete analytical workflow, including sample preparation, reagent consumption, energy requirements, waste generation, and analyst safety^47^. The proposed HDDR method achieved a MoGAPI score of 92%, compared with 80% for the reported chromatographic methods^11,12^. This result demonstrates the environmental advantages of the proposed approach and reflects its simplified sample handling, reduced reagent consumption, and minimized waste production. Furthermore, the avoidance of hazardous organic solvents contributes to improved operational safety and greenness (Table 5).

#### The analytical green star area (AGSA)

AGSA is a recently developed green metric that provides a unique visual-numerical hybrid evaluation by calculating the total area within a star-shaped pictogram. Each vertex of the star represents a core GAC principle. This systematic approach evaluates and weighs factors such as reagent toxicity, waste minimization, energy efficiency, and operator safety. The resulting star chart provides a clear visual representation of how well a method complies with each principle, allowing for an immediate assessment of the method's balance^48^. The proposed method achieved an AGSA score of 76, exceeding the values of the reported methods^11,12^. This result indicates stronger compliance with green analytical principles related to reagent safety, energy efficiency, and operational simplicity and safety (Table 5).

#### Carbon footprint reduction index (CaFRI)

CaFRI is a specialized tool designed to estimate greenhouse gas emissions associated with laboratory procedures. It assigns a numerical rating based on direct CO_2_ emission factors such as energy efficiency and indirect factors like sample storage, transportation, waste management, and reagent use^49^. In this study, our proposed spectrophotometric methods achieved an exceptional CaFRI score of 91 while the reported HPLC methods^11,12^ scored 69 and 75. As shown in Table 5, This improvement can be attributed to lower energy consumption, elimination of chromatographic separation, and reduced solvent usage. Together, these factors contribute to a more carbon-efficient analytical workflow and support the environmental sustainability of the proposed method.

### Whiteness evaluation

The concept of sustainable development has recently become a cornerstone in analytical chemistry, aligning with quality by design (QbD) principles to ensure methodological reliability alongside environmental and practical efficiency^40,42,50–53^. To evaluate these multi-faceted attributes, the red–green–blue (RGB) additive model was applied based on white analytical chemistry (WAC) principles, categorizing method features into three core pillars: redness (analytical performance), greenness (eco-friendliness), and blueness (practical and economic viability)^12,41,54–56^.

**Fig. 8:**
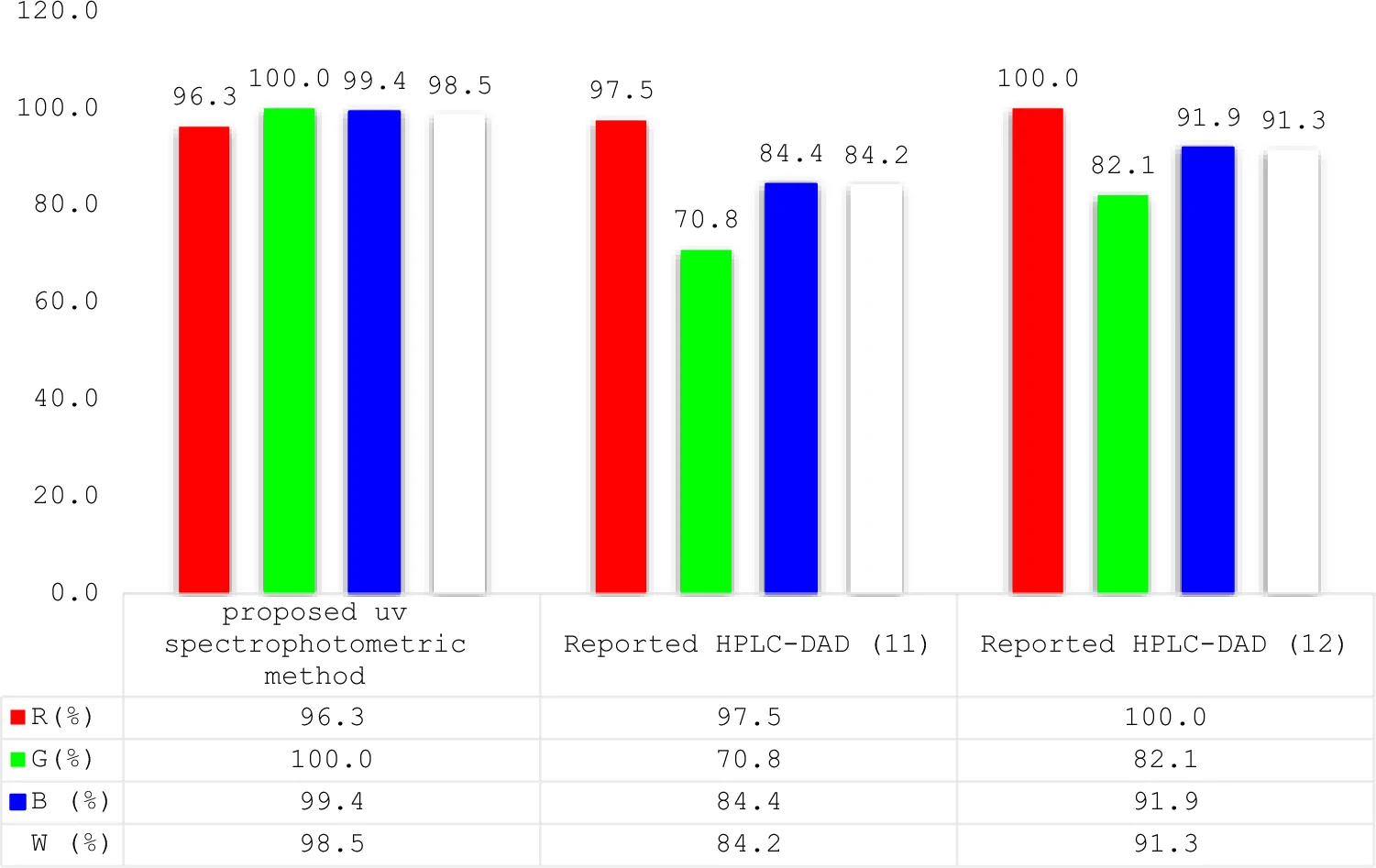
Evaluation of the degree of whiteness of the proposed spectrophotometric method in comparison with the reported HPLC-DAD methods^11,12^ using RGB algorithm.

As illustrated in Fig. 8 and Table S4, the proposed UV spectrophotometric method achieved an exceptional overall Whiteness score of 98.5%, noticeably outperforming reported HPLC–DAD methods^11,12^ which scored 84.2% and 91.3%, respectively. Regarding the Redness profile, the proposed method maintained high analytical quality with a score of 96.3% (covering scope, sensitivity, precision, and accuracy), providing reliability comparable to chromatographic benchmarks without requiring physical separation. In the greenness profile, the proposed method attained a perfect 100.0% score by eliminating toxic reagents and minimizing energy and waste, markedly surpassing reported HPLC methods (70.8% and 82.1%). Furthermore, the Blueness profile scored 99.4%, demonstrating superior cost and time efficiency as well as operational simplicity over reported HPLC methods (84.4% and 91.9%). Overall, embedding QbD principles within this WAC assessment confirms that the proposed method delivers a well-balanced, eco-friendly, and cost-effective analytical tool for routine quality control.

### Blueness evaluation

​ Blue applicability grade index (BAGI) was implemented to quantitatively evaluate the practical feasibility and suitability of analytical methodologies for routine applications. This metric provides a structured assessment of a method's “blueness” by examining ten critical operational parameters, including type of analysis, number of analytes, type of analytical technique, sample throughput, needs of sample preparation, number of samples /h, required reagents/materials, preconcentration requirement, degree of automation, and amount of sample. Each of these ten variables is assigned a numerical value ranging from 2.5 (lowest performance) to 10 (optimal performance), which corresponds to a color gradient from white to dark blue. The results are presented as an asteroid pictogram with a distinct blue shade, the darker the blue, the greater is the compliance of the method with the set of criteria^12,56–59^. The developed method achieved a higher score than the reported methods^11,12^ due to the substantial enhancement in sample throughput. In this factor, our proposed method achieved a score of 10 (dark blue), compared with scores of 5 (light blue) and 2.5 (white) for the reported methods (Table 6).

**Table 6:**
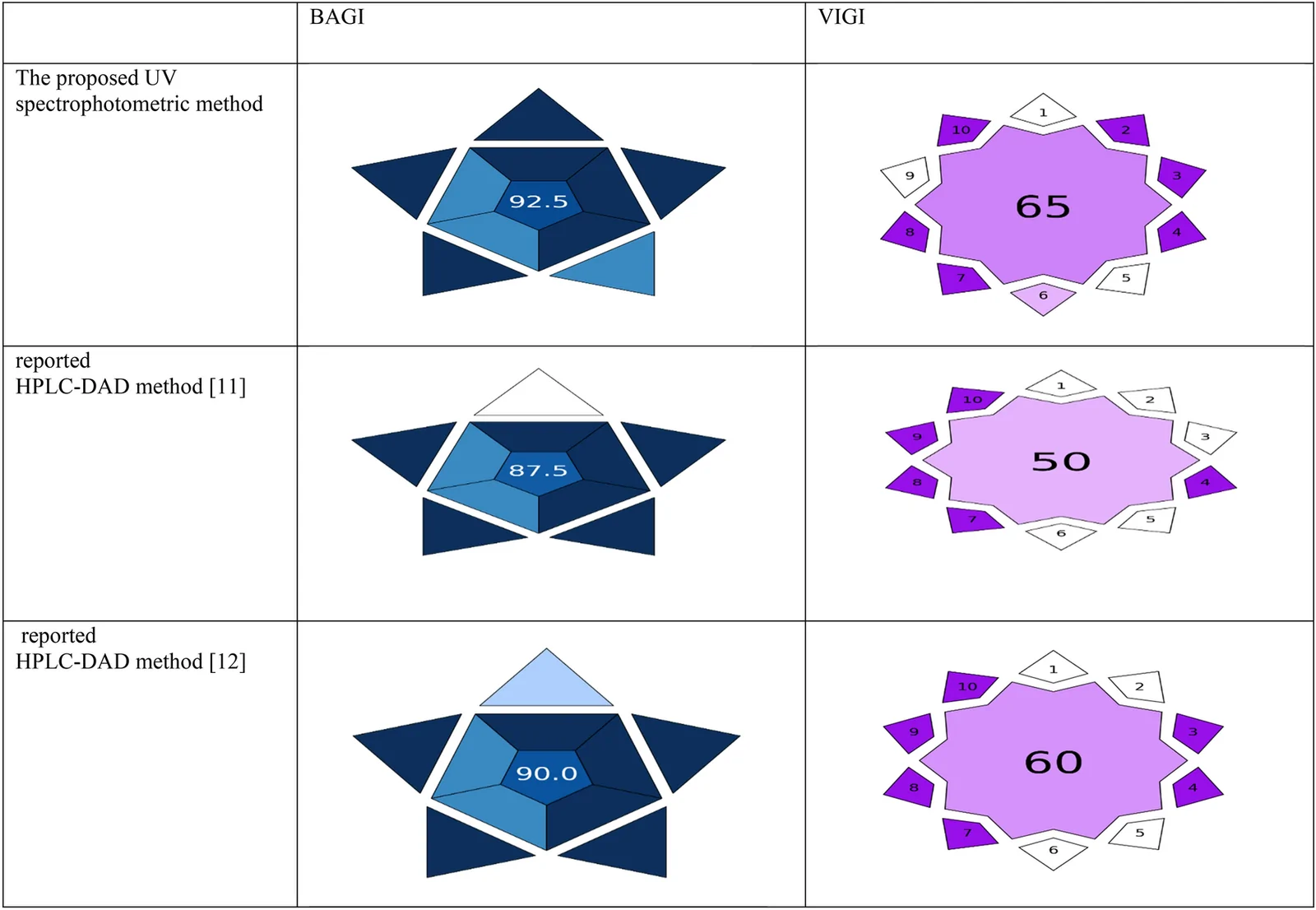
Evaluation of the degree of blueness and innovation of the proposed spectrophotometric method in comparison with the reported HPLC–DAD methods^11,12^ using BAGI and VIGI metrics.

### Violet innovation grade index (VIGI)

The innovative character of the proposed HDDR method was evaluated using the violet innovation grade index (VIGI), a survey-based metric that assesses methodological innovation through criteria related to analytical development, advanced data processing, automation, and sustainability integration^60^. The proposed method achieved a VIGI score of 65, compared with scores of 50 and 60 for the reported methods^11,12^. This score reflects the application of HDDR-based mathematical processing for the direct resolution of severely overlapping ternary spectra together with the integration of a comprehensive sustainability assessment framework. The combination of analytical performance, mathematical signal resolution, and sustainability-oriented evaluation contributed to the innovative profile of the proposed method (Table 6).

## Conclusion

A spectrophotometric strategy was developed for the simultaneous determination of OXA, 5-FU, and LU in the FOLFOX regimen. Evaluation of zero-order and first-derivative spectrophotometric approaches showed that direct spectrophotometry enabled the determination of LU, whereas first-derivative spectrophotometry allowed the determination of 5-FU and LU but was insufficient for the complete resolution of the ternary mixture. In contrast, the HDDR method successfully resolved the highly overlapping spectra of OXA, 5-FU, and LU and enabled their simultaneous quantification without prior separation.

The developed HDDR method was validated according to ICH guidelines^20^ and demonstrated satisfactory linearity, accuracy, precision, specificity, and robustness across the studied concentration ranges. Application of the method to laboratory-prepared mixtures, including mixtures representing therapeutic dose ratios of the FOLFOX regimen, confirmed its suitability for the analysis of the three drugs in the presence of one another.

The scope of the present study was the development and validation of an HDDR-based spectrophotometric method for routine quality-control analysis of the FOLFOX regimen. Future investigations may explore the applicability of this approach to more complex sample matrices.

In addition, multi-metric assessment using AES, AGREE, MoGAPI, AGSA, CaFRI, RGB, BAGI, and VIGI provided favorable scores relative to the reported chromatographic methods, reflecting the use of an aqueous solvent system, reduced waste generation, and simplified analytical workflow. The results obtained indicate that the HDDR approach is suitable for routine quality-control analysis of the FOLFOX regimen and represents an alternative spectrophotometric tool for the analysis of highly overlapping ternary mixtures.

## Supplementary Information

Supplementary Information.

## Data Availability

All data generated or analysed during this study are included in this published article and its supplementary information files.

## Abbreviations

5-FU: 5-Fluorouracil
AES: Analytical eco-scale
AGREE: Analytical greenness metric
AGSA: Analytical green star area
BAGI: Blue applicability grade index
CaFRI: Carbon footprint reduction index
CC: Colon cancer
D0: Zero-order absorption spectra
D1: First-derivative spectrophotometry/first-derivative spectra
DDRD: Double divisor ratio spectra derivative method
DRSZ: Derivative ratio spectra-zero-crossing
Er%: Percentage relative error
GAC: Green analytical chemistry
GAPI: Green analytical procedure index
HDDR: Hybrid double divisor ratio spectra method
HPLC: High-performance liquid chromatography
HPLC–DAD: High-performance liquid chromatography with diode-array detection
ICH: International council for harmonization
LOD: Limit of detection
LOQ: Limit of quantification
LU: Leucovorin
MLRA: Multi-wavelength linear regression analysis
MoGAPI: Modified green analytical procedure index
OS: Overall survival
OXA: Oxaliplatin
PCR: Principal component regression
PFS: Progression-free survival
PLS: Partial least squares
r: Correlation coefficient
RGB: Red–green–blue model
RSD%: Percentage relative standard deviation
SD: Standard deviation
QbD: Quality by design
UV–Vis: Ultraviolet–visible
VIGI: Violet innovation grade index
WAC: White analytical chemistry

## References

[1] Taieb, J. & Gallois, C. Adjuvant chemotherapy for stage III colon cancer. *Cancers* **12**, 2679 (2020). 32961795 10.3390/cancers12092679 PMC7564362

[2] Andre, T. et al. Current issues in adjuvant treatment of stage II colon cancer. *Ann. Surg. Oncol.* **13**, 887–898 (2006). 16614880 10.1245/ASO.2006.07.003

[3] Bagus, B. I. Gastrointestinal related symptoms on FOLFIRI regiments in resectable stage III colorectal cancer patients: a retrospective study. *Int. J. Surg.* **3**, 07–08 (2019).

[4] Hossain, M. S. et al. Colorectal cancer: a review of carcinogenesis, global epidemiology, current challenges, risk factors, preventive and treatment strategies. *Cancers* **14**, 1732 (2022). 35406504 10.3390/cancers14071732 PMC8996939

[5] Miura, K. et al. 5-FU metabolism in cancer and orally-administrable 5-FU drugs. *Cancers* **2**, 1717–1730 (2010). 24281184 10.3390/cancers2031717 PMC3837334

[6] Zittoun, J. et al. Pharmacokinetic comparison of leucovorin and levoleucovorin. *Eur. J. Clin. Pharmacol.* **44**, 569–573 (1993). 8405015 10.1007/BF02440861

[7] Sharma, G., Anghore, D., Khare, R. & Rawal, R. K. Oxaliplatin for colorectal cancer therapy: a review. *Clin. Cancer Drugs* **5**, 13–27 (2018).

[8] André, T. et al. Improved overall survival with oxaliplatin, fluorouracil, and leucovorin as adjuvant treatment in stage II or III colon cancer in the MOSAIC trial. *J. Clin. Oncol.* **27**, 3109–3116 (2009). 19451431 10.1200/JCO.2008.20.6771

[9] de Gramont, A. et al. Leucovorin and fluorouracil with or without oxaliplatin as first-line treatment in advanced colorectal cancer. *J. Clin. Oncol.* **41**, 5080–5089 (2023). 37967516 10.1200/JCO.22.02773

[10] BC Agency. *BC Cancer Agency Guideline* **8** (2020).

[11] Al Sabbagh, C., Agapova, E., Boudy, V. & Mignet, N. Stability of calcium levofolinate, 5-fluorouracil and oxaliplatin (FOLFOX) mixture. *J. Oncol. Pharm. Pract.* **28**, 1303–1314 (2022). 34053358 10.1177/10781552211020808

[12] Maher, H. M., Mohamed, S. M., Hassan, E. M. & El-Yazbi, A. F. Sustainability-based comparative stability of oxaliplatin plus leucovorin and 5-fluorouracil in infusion bags with application to plasma and colonic media samples. *Sci. Rep.* **15**, 17982 (2025). 40410228 10.1038/s41598-025-02079-8 PMC12102374

[13] Agrawal, A. Chemometric approaches to improve greenness. In *Sustain. Anal. Chem. Methods Mater. Appl.* 193–226 (2026).

[14] Wahbi, A. A., Mabrouk, M. M., Moneeb, M. S. & Kamal, A. H. Simultaneous determination of the two non-steroidal anti-inflammatory drugs; diflunisal and naproxen in their tablets by chemometric spectrophotometry and HPLC. *Pak. J. Pharm. Sci.* **22**, 8–17 (2009). 19168413

[15] Korany, M. A., Maher, H. M., Galal, S. M., Fahmy, O. T. & Ragab, M. A. Non-parametric linear regression of discrete Fourier transform convoluted chromatographic peak responses under non-ideal conditions of internal standard method. *Talanta* **83**, 93–109 (2010). 21035649 10.1016/j.talanta.2010.08.046

[16] Maher, H. M., Ragab, M. A. A. & El-Kimary, E. I. Chemometrics-assisted spectrofluorimetric determination of two co-administered drugs of major interaction, methotrexate and aspirin, in human urine following acid-induced hydrolysis. *Comb. Chem. High Throughput Screen.* **18**, 723–734 (2015). 26234512 10.2174/1386207318666150803140749

[17] Maher, H. M. Recent advances in applications of capillary electrophoresis with Fourier transform convolution: application to kinetic study of hydrolysis of hydrochlorothiazide. *Biomed. Chromatogr.* **28**, 573–582 (2014).

[18] Maher, H. M., Mahgoub, H., Ragab, M. A. & Tarek, S. Green Chemometric—assisted UV spectrophotometric techniques for the analysis of Helicobacter pylori triple therapy: amoxicillin, metronidazole and famotidine in bulk, individual and laboratory-prepared combined dosage forms: application to simulated gastric fluid with comprehensive greenness and whiteness appraisals. *BMC Chem.* **19**, 44 (2025). 39972462 10.1186/s13065-025-01387-4 PMC11841278

[19] Youssef, R. M. & Maher, H. M. A new hybrid double divisor ratio spectra method for the analysis of ternary mixtures. *Spectrochim. Acta A Mol. Biomol. Spectrosc.* **70**, 1152–1166 (2008). 18093870 10.1016/j.saa.2007.10.049

[20] International Conference on Harmonisation (ICH).* Validation of Analytical Procedures: Text and Methodology* Q2(R1) (2005).

[21] Giese, A. T. & French, C. S. The analysis of overlapping spectral absorption bands by derivative spectrophotometry. *Appl. Spectrosc.* **9**, 78–96 (1955).

[22] Levillain, P. & Fompeydie, D. Spectrophotométrie dérivée: intérêt, limites et applications. *Analusis* **14**, 1–20 (1986).

[23] Heilmeyer, L., Jordan, A. & Tippell, T. Spectrophotometry in medicine (1943).

[24] Wahbi, M., Hassan, E., Khamis, E., Hamdy, D. & Barady, M. Generation of derivative curves of absorption spectra using Glenn's method of orthogonal functions. *J. Pharm. Pharmacol.* **5**5 (2003).

[25] Wahbi, A., Abdine, H. & Korany, M. The use of Fourier functions to eliminate interferences in spectrophotometric analysis (theory and application). *Pharmazie* **33**, 278–282 (1978). 674337

[26] Aboras, S. I., Abdelhakim, M. R., Maher, H. M. & Youssef, R. M. Cutting-edge assays for mirabegron and tadalafil combo therapy for benign prostatic hyperplasia; insilico kinetics approach; multi trait sustainability assessment. *BMC Chem.* **19**, 140 (2025). 40410841 10.1186/s13065-025-01497-z PMC12101028

[27] Wahbi, A., Abdine, H., Korany, M. & El-Yazbi, F. Spectrophotometric analysis of binary mixtures of antazoline and naphazoline. *J. Pharm. Sci.* **67**, 140–141 (1978). 619107 10.1002/jps.2600670143

[28] Dinç, E., Yücesoy, C. & Onur, F. Simultaneous spectrophotometric determination of mefenamic acid and paracetamol in a pharmaceutical preparation using ratio spectra derivative spectrophotometry and chemometric methods. *J. Pharm. Biomed. Anal.* **28**, 1091–1100 (2002). 12049974 10.1016/s0731-7085(02)00031-6

[29] Moneeb, M. S. Polarographic chemometric determination of zinc and nickel in aqueous samples. *Talanta* **70**, 1035–1043 (2006). 18970878 10.1016/j.talanta.2006.02.016

[30] Blanco, M., Gene, J., Iturriaga, H., Maspoch, S. & Riba, J. Diode-array detectors in flow-injection analysis mixture resolution by multi-wavelength analysis. *Talanta* **34**, 987–993 (1987). 18964446 10.1016/0039-9140(87)80146-7

[31] Salinas, F., Nevado, J. B. & Mansilla, A. E. A new spectrophotometric method for quantitative multicomponent analysis resolution of mixtures of salicylic and salicyluric acids. *Talanta* **37**, 347–351 (1990). 18964949 10.1016/0039-9140(90)80065-n

[32] Nevado, J. B., Cabanillas, C. G. & Salinas, F. Spectrophotometric resolution of ternary mixtures of salicylaldehyde, 3-hydroxybenzaldehyde and 4-hydroxybenzaldehyde by the derivative ratio spectrum-zero crossing method. *Talanta* **39**, 547–553 (1992). 18965415 10.1016/0039-9140(92)80179-h

[33] Sinha, V., Kumar, R. & Bhinge, J. A stability-indicating RP-HPLC assay method for 5-fluorouracil. *Indian J. Pharm. Sci.* **71**, 630 (2009). 20376215 10.4103/0250-474X.59544 PMC2846467

[34] Jerremalm, E. et al. Alkaline hydrolysis of oxaliplatin—isolation and identification of the oxalato monodentate intermediate. *J. Pharm. Sci.* **91**, 2116–2121 (2002). 12226839 10.1002/jps.10201

[35] Verstraete, S., Heudi, O., Cailleux, A. & Allain, P. Comparison of the reactivity of oxaliplatin, Pt(diaminocyclohexane)Cl_2_ and Pt(diaminocyclohexane1)(OH_2_)_2_^(2+)^ with guanosine and L-methionine. *J. Inorg. Biochem.* **84**, 129–135 (2001). 11330471 10.1016/s0162-0134(00)00208-7

[36] Mehta, A. M. et al. Stability of oxaliplatin in chloride-containing carrier solutions used in hyperthermic intraperitoneal chemotherapy. *Int. J. Pharm.* **479**, 23–27 (2015). 25535649 10.1016/j.ijpharm.2014.12.025

[37] Cosulich, D. B., Roth, B., Smith, J. M. Jr., Hultquist, M. E. & Parker, R. P. Chemistry of leucovorin. *J. Am. Chem. Soc.* **74**, 3252–3263 (1952).

[38] Lévi, F., Metzger, G., Massari, C. & Milano, G. Oxaliplatin: Pharmacokinetics and chronopharmacological aspects. *Clin. Pharmacokinet.* **38**, 1–21 (2000). 10668856 10.2165/00003088-200038010-00001

[39] Miller, J. N. & Miller, J. C. *Statistics and Chemometrics for Analytical Chemistry* 5th ed. (Prentice Hall, 2005).

[40] Nuli, M. V. et al. Sustainable quality by design approach based UHPLC technique for triple combination antiviral drugs: appraisal of green and white metrics. *Sep. Sci. Plus* **8**, e70085 (2025).

[41] Prajapati, P. et al. Microwave-assisted fluorodensitometric estimation of baclofen using quality by design and white analytical chemistry approach. *JPC – J. Planar Chromatogr. – Modern TLC* **38**, 155–173 (2025).

[42] Prajapati, P., Prajapati, B., Haque, A., Ahmad, S. & Shah, S. Failure mode effects analysis and design of experiments to sensitive and sustainable microwave-aided spectrofluorophotometric estimation of teneligliptin and method's chlor-tox scale and blue applicability grade index assessment. *Luminescence* **40**, e70156 (2025). 40170631 10.1002/bio.70156

[43] Prajapati, P. et al. Eco-friendly nano-scale bio-analytical insights for spectrofluorimetric estimation of fimasartan using integrated approach of enhanced microwave-assisted Hantzsch reaction and multicolored analytical chemistry. *J. Fluoresc.* **35**, 12665–12678 (2025). 40818015 10.1007/s10895-025-04514-5

[44] Prajapati, P., Haque, A., Kalam, M. A. & Shah, S. Microwave-derivatized enhanced-spectrofluorophotometric characterization of nateglinide-loaded solid dispersion adsorbate and greenness profile assessment of method. *Anal. Sci.* **41**, 263–279 (2025). 39648264 10.1007/s44211-024-00694-5

[45] Tobiszewski, M., Marć, M., Gałuszka, A. & Namieśnik, J. Green chemistry metrics with special reference to green analytical chemistry. *Molecules* **20**, 10928–10946 (2015). 26076112 10.3390/molecules200610928 PMC6272361

[46] Pena-Pereira, F., Wojnowski, W. & Tobiszewski, M. AGREE—Analytical greenness metric approach and software. *Anal. Chem.* **92**, 10076–10082 (2020). 32538619 10.1021/acs.analchem.0c01887 PMC7588019

[47] Mansour, F. R., Płotka-Wasylka, J. & Locatelli, M. Modified GAPI (MoGAPI) tool and software for the assessment of method greenness: case studies and applications. *Analytica* **5**, 451–457 (2024).

[48] Mansour, F. R., Bedair, A., Belal, F., Magdy, G. & Locatelli, M. Analytical green star area (AGSA) as a new tool to assess greenness of analytical methods. *Sustain. Chem. Pharm.* **46**, 102051 (2025).

[49] Mansour, F. R. & Nowak, P. M. Introducing the carbon footprint reduction index (CaFRI) as a software-supported tool for greener laboratories in chemical analysis. *BMC Chem.* **19**, 121 (2025). 40346688 10.1186/s13065-025-01486-2 PMC12065229

[50] Waghmare, S. et al. Sustainable and stability-indicating RP-HPLC method for Tofacitinib: an integrated white analytical chemistry and A-QbD approach. *Discov. Chem.* **3**, 143 (2026).

[51] Sawale, V., Sabale, P., Kunte, R. & Sabale, V. Development of eco-friendly and sustainable analytical methods for Ritonavir through white analytical chemistry and AQbD approach. *Discov. Chem.* **3**, 151 (2026).

[52] Dokewar, I. R. & Pawar, S. S. In-silico tools and analytical quality by design in liquid chromatographic method development. *J. Liq. Chromatogr. Relat. Technol.* **49**, 172–187 (2026).

[53] Prajapati, P., Nhavi, N., Haque, A., Ahmad, S. & Shah, S. Sensitive and selective fluorescent Mannich adduct biosensing probe for estimation of Carvedilol using integrated approach of white analytical chemistry and quality by design. *J. Pharm. Innov.* **21**, 141 (2026).

[54] Nowak, P. M. & Kościelniak, P. What color is your method? Adaptation of the RGB additive color model to analytical method evaluation. *Anal. Chem.* **91**, 10343–10352 (2019). 31305064 10.1021/acs.analchem.9b01872

[55] Nowak, P. M., Wietecha-Posłuszny, R. & Pawliszyn, J. White analytical chemistry: an approach to reconcile the principles of green analytical chemistry and functionality. *Trends Anal. Chem.* **138**, 116223 (2021).

[56] Prajapati, P. B. et al. Blue applicability grade index assessment and response surface modelling to synchronous determination of metformin hydrochloride, vildagliptin and dapagliflozin propanediol monohydrate by HPTLC method. *Future J. Pharm. Sci.* **11** (2025).

[57] Manousi, N., Wojnowski, W., Płotka-Wasylka, J. & Samanidou, V. Blue applicability grade index (BAGI) and software: a new tool for the evaluation of method practicality. *Green Chem.* **25**, 7598–7604 (2023).

[58] El-Shaheny, R. et al. Green spectrofluorimetric method using nitrogen–phosphorus carbon quantum dots and Box–Behnken optimized salting-out assisted extraction for valbenazine determination in environmental samples. *Sci. Rep.* **16** (2026). 10.1038/s41598-026-63824-1 PMC13408159 42509264

[59] Kannaiah, K., Sharma, H., Kumar, H., Keshishian, R. & Obaydo, R. Analytical methods for bexarotene determination: a comparative review of chromatographic techniques through the lens of green chemistry. *Green Chem. Lett. Rev.* **19** (2026).

[60] Fuente-Ballesteros, A. et al. Violet innovation grade index (VIGI): A new survey-based metric for evaluating innovation in analytical methods. *Anal. Chem.* **97**, 6946–6955 (2025). 40139928 10.1021/acs.analchem.5c00212 PMC12131222

