# Supplementary material for: Fourier-based chemometric resolution of the FOLFOX regimen through digital filtering with poly-metric sustainability assessment
Source: Sci Rep. 2026 Sep 11;16:28487. doi: 10.1038/s41598-026-67979-9 (PMC13569837; doi:10.1038/s41598-026-67979-9)
Supplement: Supplementary file 1 [file 41598_2026_67979_MOESM1_ESM.docx]

Section S1

Mathematical Sequence of the Hybrid Double Divisor Ratio Spectra Method (HDDR)^19^

If a ternary mixture of three analytes, $X, Y$ and$Z$, is considered and if the absorbance value of this ternary mixture is measured at$\lambda i$, the following equation can be written as

$\boldsymbol{A}_{m,\lambda i}\boldsymbol{=}\boldsymbol{\alpha}_{\left\{ \boldsymbol{X}\boldsymbol{,}\boldsymbol{\lambda} \right\}}\boldsymbol{C}_{\boldsymbol{X}}\boldsymbol{+}\boldsymbol{\beta}_{\left\{ \boldsymbol{Y}\boldsymbol{,}\boldsymbol{\lambda} \right\}}\boldsymbol{C}_{\boldsymbol{Y}}\boldsymbol{+}\boldsymbol{\gamma}_{\left\{ \boldsymbol{Z}\boldsymbol{,}\boldsymbol{\lambda} \right\}}\boldsymbol{C}_{\boldsymbol{Z}}$	(1)

where Am,λi is the absorbance of the mixture at wavelength $\lambda i$ and$\boldsymbol{\alpha}_{\left\{ \boldsymbol{X,\lambda} \right\}\boldsymbol{,}}\boldsymbol{\beta}_{\left\{ \boldsymbol{Y,\lambda} \right\}}$ $\mathrm{and} \boldsymbol{\gamma}_{\left\{ \boldsymbol{Z,\lambda} \right\}}$are the absorptivities of $X, Y$ and$Z$, respectively. $\boldsymbol{C}_{\boldsymbol{X}}$, $\boldsymbol{C}_{\boldsymbol{Y}}$ and $\boldsymbol{C}_{\boldsymbol{Z}}$ represent the concentrations of compounds.

A similar equation for two compounds in the same ternary mixture as in a standard binary mixture can be written as

$\boldsymbol{A}_{m,\lambda i}\boldsymbol{=}\boldsymbol{\alpha}_{\left\{ \boldsymbol{X}\boldsymbol{,}\boldsymbol{\lambda} \right\}}\boldsymbol{C}_{\boldsymbol{X}}^{\boldsymbol{0}}\boldsymbol{+}\boldsymbol{\beta}_{\left\{ \boldsymbol{Y}\boldsymbol{,}\boldsymbol{\lambda} \right\}}\boldsymbol{C}_{\boldsymbol{Y}}^{\boldsymbol{0}}$	(2)

If Eq. (1) is divided by Eq. (2) corresponding to the spectrum of a standard solution of two of the components in the ternary mixture, the ratio spectrum is obtained in the form of Eq. (3)

$\frac{\boldsymbol{A}_{\left\{ \boldsymbol{m}\boldsymbol{,}\boldsymbol{\lambda} \right\}}}{\boldsymbol{\alpha}_{\left\{ \boldsymbol{X}\boldsymbol{,}\boldsymbol{\lambda} \right\}}\boldsymbol{C}_{\boldsymbol{X}}^{\boldsymbol{0}}\boldsymbol{+}\boldsymbol{\beta}_{\left\{ \boldsymbol{Y}\boldsymbol{,}\boldsymbol{\lambda} \right\}}\boldsymbol{C}_{\boldsymbol{Y}}^{\boldsymbol{0}}}\boldsymbol{=}\frac{\boldsymbol{\alpha}_{\left\{ \boldsymbol{X}\boldsymbol{,}\boldsymbol{\lambda} \right\}}\boldsymbol{C}_{\boldsymbol{X}}}{\boldsymbol{\alpha}_{\left\{ \boldsymbol{X}\boldsymbol{,}\boldsymbol{\lambda} \right\}}\boldsymbol{C}_{\boldsymbol{X}}^{\boldsymbol{0}}\boldsymbol{+}\boldsymbol{\beta}_{\left\{ \boldsymbol{Y}\boldsymbol{,}\boldsymbol{\lambda} \right\}}\boldsymbol{C}_{\boldsymbol{Y}}^{\boldsymbol{0}}}\boldsymbol{+}\frac{\boldsymbol{\beta}_{\left\{ \boldsymbol{Y}\boldsymbol{,}\boldsymbol{\lambda} \right\}}\boldsymbol{C}_{\boldsymbol{Y}}}{\boldsymbol{\alpha}_{\left\{ \boldsymbol{X}\boldsymbol{,}\boldsymbol{\lambda} \right\}}\boldsymbol{C}_{\boldsymbol{X}}^{\boldsymbol{0}}\boldsymbol{+}\boldsymbol{\beta}_{\left\{ \boldsymbol{Y}\boldsymbol{,}\boldsymbol{\lambda} \right\}}\boldsymbol{C}_{\boldsymbol{Y}}^{\boldsymbol{0}}}\boldsymbol{+}\frac{\boldsymbol{\gamma}_{\left\{ \boldsymbol{Z}\boldsymbol{,}\boldsymbol{\lambda} \right\}}\boldsymbol{C}_{\boldsymbol{Z}}}{\boldsymbol{\alpha}_{\left\{ \boldsymbol{X}\boldsymbol{,}\boldsymbol{\lambda} \right\}}\boldsymbol{C}_{\boldsymbol{X}}^{\boldsymbol{0}}\boldsymbol{+}\boldsymbol{\beta}_{\left\{ \boldsymbol{Y}\boldsymbol{,}\boldsymbol{\lambda} \right\}}\boldsymbol{C}_{\boldsymbol{Y}}^{\boldsymbol{0}}}$

$\frac{\boldsymbol{A}_{\left\{ \boldsymbol{m,\lambda} \right\}}}{\boldsymbol{\alpha}_{\left\{ \boldsymbol{X,\lambda} \right\}}\boldsymbol{C}_{\boldsymbol{X}}^{\boldsymbol{0}}\boldsymbol{+}\boldsymbol{\beta}_{\left\{ \boldsymbol{Y,\lambda} \right\}}\boldsymbol{C}_{\boldsymbol{Y}}^{\boldsymbol{0}}}\boldsymbol{=}\frac{\boldsymbol{\alpha}_{\left\{ \boldsymbol{X,\lambda} \right\}}\boldsymbol{C}_{\boldsymbol{X}}\boldsymbol{+}\boldsymbol{\beta}_{\left\{ \boldsymbol{Y,\lambda} \right\}}\boldsymbol{C}_{\boldsymbol{Y}}}{\boldsymbol{\alpha}_{\left\{ \boldsymbol{X,\lambda} \right\}}\boldsymbol{C}_{\boldsymbol{X}}^{\boldsymbol{0}}\boldsymbol{+}\boldsymbol{\beta}_{\left\{ \boldsymbol{Y,\lambda} \right\}}\boldsymbol{C}_{\boldsymbol{Y}}^{\boldsymbol{0}}}\boldsymbol{+}\frac{\boldsymbol{\gamma}_{\left\{ \boldsymbol{Z,\lambda} \right\}}\boldsymbol{C}_{\boldsymbol{Z}}}{\boldsymbol{\alpha}_{\left\{ \boldsymbol{X,\lambda} \right\}}\boldsymbol{C}_{\boldsymbol{X}}^{\boldsymbol{0}}\boldsymbol{+}\boldsymbol{\beta}_{\left\{ \boldsymbol{Y,\lambda} \right\}}\boldsymbol{C}_{\boldsymbol{Y}}^{\boldsymbol{0}}}$	(3)

The ratio of the sum of $\alpha_{\left\{ X,\lambda\right\}}C_{X} \mathrm{and} \beta_{\left\{ Y,\lambda\right\}}C_{Y}$ to the sum of $\alpha_{\left\{ X,\lambda\right\}}C_{X}^{0}\mathrm{and} \beta_{\left\{ Y,\lambda\right\}}C_{Y}^{0}$is equal to a constant (k) with respect to λ, in a certain region or point of wavelength (Fig.2) and if the above constant is replaced in Eq. (3), we obtain Eq. (4):

$\frac{\boldsymbol{A}_{\left\{ \boldsymbol{m}\boldsymbol{,}\boldsymbol{\lambda} \right\}}}{\boldsymbol{\alpha}_{\left\{ \boldsymbol{X}\boldsymbol{,}\boldsymbol{\lambda} \right\}}\boldsymbol{C}_{\boldsymbol{X}}^{\boldsymbol{0}}\boldsymbol{+}\boldsymbol{\beta}_{\left\{ \boldsymbol{Y}\boldsymbol{,}\boldsymbol{\lambda} \right\}}\boldsymbol{C}_{\boldsymbol{Y}}^{\boldsymbol{0}}}\boldsymbol{=}\boldsymbol{K} \left( \boldsymbol{Constant} \right)\boldsymbol{+}\frac{\boldsymbol{\gamma}_{\left\{ \boldsymbol{Z}\boldsymbol{,}\boldsymbol{\lambda} \right\}}\boldsymbol{C}_{\boldsymbol{Z}}}{\boldsymbol{\alpha}_{\left\{ \boldsymbol{X}\boldsymbol{,}\boldsymbol{\lambda} \right\}}\boldsymbol{C}_{\boldsymbol{X}}^{\boldsymbol{0}}\boldsymbol{+}\boldsymbol{\beta}_{\left\{ \boldsymbol{Y}\boldsymbol{,}\boldsymbol{\lambda} \right\}}\boldsymbol{C}_{\boldsymbol{Y}}^{\boldsymbol{0}}}$	(4)

However, if the standard concentrations of $C_{X}^{0}$ and $C_{Y}^{0}$ in Eq. (5) are equal or very close to each other, ($C_{X}^{0}$ = $C_{Y}^{0}$) or ($C_{X}^{0}$≈ $C_{Y}^{0}$) we could write:

$\boldsymbol{\alpha}_{\left\{ \boldsymbol{X}\boldsymbol{,}\boldsymbol{\lambda}_{\boldsymbol{i}} \right\}}\boldsymbol{C}_{\boldsymbol{X}}^{\boldsymbol{0}}\boldsymbol{+}\boldsymbol{\beta}_{\left\{ \boldsymbol{Y}\boldsymbol{,}\boldsymbol{\lambda}_{\boldsymbol{i}} \right\}}\boldsymbol{C}_{\boldsymbol{Y}}^{\boldsymbol{0}}\boldsymbol{=}\boldsymbol{C}_{\boldsymbol{X}}^{\boldsymbol{0}}\left[ \boldsymbol{\alpha}_{\left\{ \boldsymbol{X}\boldsymbol{,}\boldsymbol{\lambda}_{\boldsymbol{i}} \right\}}\boldsymbol{+}\boldsymbol{\beta}_{\left\{ \boldsymbol{Y}\boldsymbol{,}\boldsymbol{\lambda}_{\boldsymbol{i}} \right\}} \right]$	(5)

When Eq. (5) is substituted into Eq. (4), Eq. (6) is obtained

$\frac{\boldsymbol{A}_{\left\{ \boldsymbol{m}\boldsymbol{,}\boldsymbol{\lambda} \right\}}}{\boldsymbol{C}_{\boldsymbol{X}}^{\boldsymbol{0}}\left[ \boldsymbol{\alpha}_{\left\{ \boldsymbol{X}\boldsymbol{,}\boldsymbol{\lambda}_{\boldsymbol{i}} \right\}}\boldsymbol{+}\boldsymbol{\beta}_{\left\{ \boldsymbol{Y}\boldsymbol{,}\boldsymbol{\lambda}_{\boldsymbol{i}} \right\}} \right]}\boldsymbol{=}\boldsymbol{K} \left( \boldsymbol{Constant} \right)\boldsymbol{+}\frac{\boldsymbol{\gamma}_{\left\{ \boldsymbol{Z}\boldsymbol{,}\boldsymbol{\lambda} \right\}}\boldsymbol{C}_{\boldsymbol{Z}}}{\boldsymbol{C}_{\boldsymbol{X}}^{\boldsymbol{0}}\left[ \boldsymbol{\alpha}_{\left\{ \boldsymbol{X}\boldsymbol{,}\boldsymbol{\lambda}_{\boldsymbol{i}} \right\}}\boldsymbol{+}\boldsymbol{\beta}_{\left\{ \boldsymbol{Y}\boldsymbol{,}\boldsymbol{\lambda}_{\boldsymbol{i}} \right\}} \right]}$	(6)

According to the basic principle for the use of the trigonometric Fourier functions to process absorbance ratio spectra , an absorbance ratio spectrum can be expanded in terms of the Fourier series as follows ^15^:

$$\boldsymbol{A}_{\boldsymbol{r}\left( \boldsymbol{\lambda} \right)}\boldsymbol{=}\boldsymbol{a}_{\boldsymbol{0}}\boldsymbol{+}\boldsymbol{a}_{\boldsymbol{1}}\cos\boldsymbol{x}\boldsymbol{+}\boldsymbol{a}_{\boldsymbol{2}}\cos\boldsymbol{2}\boldsymbol{x}\boldsymbol{+}\boldsymbol{a}_{\boldsymbol{3}}\cos\boldsymbol{3}\boldsymbol{x}\boldsymbol{+ \ldots+}\boldsymbol{b}_{\boldsymbol{1}}\sin\boldsymbol{x}\boldsymbol{+}\boldsymbol{b}_{\boldsymbol{2}}\sin\boldsymbol{2}\boldsymbol{x}\boldsymbol{+}\boldsymbol{b}_{\boldsymbol{3}}\sin\boldsymbol{3}\boldsymbol{x}\boldsymbol{+ \ldots}$$

(7)

where Ar($\lambda$) denotes the absorbance ratio, obtained by using a double divisor, at a set of (n + 1) equally spaced wavelengths, aj are the coefficients of cos $jx$ and bj are the coefficients of sin $jx$ ($j$= 1, 2, 3, ..., n). $\boldsymbol{a}_{\boldsymbol{0}}$ is the constant component, $\boldsymbol{a}_{\boldsymbol{1}}$cos $\boldsymbol{x}$ is the quadratic component, etc. of the absorbance ratio gross curve. A linear component is not present in the above expansion, however a combination of two trigonometric functions has been proved to correct for this ^21^. In view of the orthogonality property of the Fourier functions, contribution from any components other than the specified compound can be automatically eliminated by convoluting the double divisor ratio spectrum, Ar($\lambda$), obtained for the analyte with a suitably selected combined trigonometric Fourier functions simply for any number of points greater than 2 (6, 8 or 10 points are usually selected)^21^.

The suitable function is selected according to the shape of the double divisor ratio spectrum. The optimum number of points and wavelength interval are selected for a given component at which the calculated Fourier coefficient should afford precise and accurate estimate of that analyte. Any coefficient of the combined trigonometric Fourier function-double divisor ratio spectrum,${t’}_{\mathrm{rj}}$ , can be calculated from a set of $\left( n + 1 \right)$ absorbance ratios measured at equally spaced wavelengths, by the following summation, in which $x$ takes values from 0 to $2\pi- \left[ \frac{2\pi}{n + 1} \right]$, at intervals of $\frac{2\pi}{n + 1}$:

$\boldsymbol{t}_{\boldsymbol{rj}}^{\boldsymbol{'}}\boldsymbol{=}\sum_{\boldsymbol{i}\boldsymbol{=}\boldsymbol{0}}^{\boldsymbol{n}} \frac{\boldsymbol{A}_{\boldsymbol{ri}}{\boldsymbol{T}\boldsymbol{’}}_{\boldsymbol{ji}}}{\boldsymbol{D}}$	(8)

where T represents the combined Fourier functions whose order j = 1, 2, 3, ..., n.

$\boldsymbol{T’}_{\boldsymbol{i}}\boldsymbol{=}\left[ \mathbf{co}\mathbf{s} \boldsymbol{jx}\boldsymbol{+ cosj}\left( \boldsymbol{x +}\frac{\boldsymbol{2}\boldsymbol{\pi}}{\boldsymbol{n + 1}} \right) \right]$ or $\boldsymbol{T’}_{\boldsymbol{i}}\boldsymbol{=}\left[ \mathbf{si}\mathbf{n} \boldsymbol{jx}\boldsymbol{-}\mathbf{si}\mathbf{n} \boldsymbol{j}\left( \boldsymbol{x +}\frac{\boldsymbol{2}\boldsymbol{\pi}}{\boldsymbol{n + 1}} \right) \right]$.

The denominator in the above equation is mathematically defined as the sum of the squares of the combined trigonometric functions over the selected number of points .

$D\boldsymbol{=}\sum_{\boldsymbol{i=0}}^{\boldsymbol{n}} \left( \boldsymbol{T}_{\boldsymbol{i}} \right)$^2^	(9)

The optimum coefficient corresponds to a maximum or a minimum in the transformed double divisor ratio spectrum. Such spectrum is obtained by plotting the ${t’}_{rj}$coefficients versus $\lambda m$ (the mean of the wavelengths set). The optimized coefficient ${t’}_{rj}$ is proportional to the concentration of the analyte. That is

$\boldsymbol{t’}_{\boldsymbol{rj}}\boldsymbol{= \alpha C}$	(10)

where $\alpha$ is a constant and $C$ is the concentration of the analyte. In the case of multi-component mixtures, e.g. a ternary mixture of three components $X, Y$ and$Z$, the absorbance spectra of the mixtures is divided by sum of the spectra of $X \mathrm{and} Y$ as a double divisor, the obtained ratio spectra are convoluted using the selected combined trigonometric Fourier functions and the observed coefficients are then plotted against λm. In this method, the concentration $C_{Z}$ is proportional to the ${t’}_{rj}$signals in the coincided points corresponding to the maximum and minimum of wavelength for pure $Z$ and its ternary mixture with $X \mathrm{and} Y$ . The concentrations of $X \mathrm{and} Y$ are determined by analogous procedures.

A

| Wavelength (nm) | MIX | OXA 50 μg/mL | 5-FU 50μg/mL | LU 10μg/mL |
| --- | --- | --- | --- | --- |
| 200 | 2.4731 | 0.8652 | 1.645 | 0.6267 |
| 202 | 3.0192 | 1.0194 | 2.4621 | 0.6383 |
| 204 | 3.547 | 0.959 | 2.8285 | 0.5889 |
| 206 | 3.5013 | 0.9144 | 2.9572 | 0.5513 |
| 208 | 3.5726 | 0.8714 | 2.9404 | 0.5221 |
| 210 | 3.529 | 0.8147 | 2.8769 | 0.4985 |
| 212 | 3.5537 | 0.7436 | 2.7481 | 0.4815 |
| 214 | 3.3586 | 0.6643 | 2.5383 | 0.47 |
| 216 | 3.111 | 0.5814 | 2.2543 | 0.4589 |
| 218 | 2.7746 | 0.4986 | 1.934 | 0.4458 |
| 220 | 2.3948 | 0.4193 | 1.5884 | 0.4306 |
| 222 | 2.0041 | 0.3488 | 1.2612 | 0.4147 |
| 224 | 1.6656 | 0.2903 | 0.9798 | 0.3986 |
| 226 | 1.4049 | 0.2441 | 0.7683 | 0.3826 |
| 228 | 1.2149 | 0.2074 | 0.6211 | 0.3663 |
| 230 | 1.0905 | 0.1783 | 0.5345 | 0.3493 |
| 232 | 1.0308 | 0.1558 | 0.509 | 0.3324 |
| 234 | 1.0292 | 0.1389 | 0.5371 | 0.3164 |
| 236 | 1.0744 | 0.1269 | 0.6074 | 0.3019 |
| 238 | 1.1554 | 0.1181 | 0.7098 | 0.2886 |
| 240 | 1.2657 | 0.1116 | 0.8384 | 0.2778 |
| 242 | 1.3993 | 0.1073 | 0.9891 | 0.2692 |
| 244 | 1.5516 | 0.1043 | 1.1533 | 0.263 |
| 246 | 1.7176 | 0.1021 | 1.3266 | 0.2584 |
| 248 | 1.8925 | 0.1005 | 1.5103 | 0.2552 |
| 250 | 2.0749 | 0.0988 | 1.699 | 0.2532 |
| 252 | 2.2592 | 0.097 | 1.8854 | 0.2527 |
| 254 | 2.4251 | 0.0956 | 2.0596 | 0.253 |
| 256 | 2.5775 | 0.0941 | 2.2089 | 0.2536 |
| 258 | 2.6991 | 0.0924 | 2.3382 | 0.255 |
| 260 | 2.8118 | 0.0906 | 2.4407 | 0.2561 |
| 262 | 2.87 | 0.0883 | 2.5086 | 0.2575 |
| 264 | 2.8786 | 0.0858 | 2.5341 | 0.2584 |
| 266 | 2.842 | 0.0835 | 2.5552 | 0.2592 |
| 268 | 2.8635 | 0.0806 | 2.5278 | 0.2604 |
| 270 | 2.7933 | 0.0781 | 2.4679 | 0.2622 |
| 272 | 2.6854 | 0.0752 | 2.3722 | 0.2648 |
| 274 | 2.5466 | 0.0723 | 2.2415 | 0.2684 |
| 276 | 2.3814 | 0.0689 | 2.0723 | 0.2723 |
| 278 | 2.1809 | 0.0663 | 1.871 | 0.2767 |
| 280 | 1.9396 | 0.0639 | 1.6375 | 0.2805 |
| 282 | 1.6976 | 0.0618 | 1.3917 | 0.2834 |
| 284 | 1.4472 | 0.0592 | 1.146 | 0.2834 |
| 286 | 1.1977 | 0.0568 | 0.9014 | 0.2811 |
| 288 | 0.9608 | 0.0546 | 0.6699 | 0.2758 |
| 290 | 0.7523 | 0.0521 | 0.4701 | 0.2683 |
| 292 | 0.584 | 0.05 | 0.3126 | 0.2589 |
| 294 | 0.4536 | 0.0482 | 0.194 | 0.2478 |
| 296 | 0.3574 | 0.0462 | 0.1113 | 0.2347 |
| 298 | 0.2922 | 0.044 | 0.0588 | 0.2222 |
| 300 | 0.2522 | 0.042 | 0.0288 | 0.2119 |
| 302 | 0.2301 | 0.04 | 0.0126 | 0.2051 |
| 304 | 0.2194 | 0.038 | 0.0042 | 0.2016 |
| 306 | 0.2169 | 0.0363 | 0.0001 | 0.2017 |
| 308 | 0.2203 | 0.035 | -0.001 | 0.2049 |
| 310 | 0.2274 | 0.033 | -0.003 | 0.2102 |
| 312 | 0.2372 | 0.0314 | -0.003 | 0.2178 |
| 314 | 0.2486 | 0.0297 | -0.003 | 0.2265 |
| 316 | 0.2624 | 0.0282 | -0.003 | 0.237 |
| 318 | 0.2771 | 0.0265 | -0.003 | 0.2484 |
| 320 | 0.2923 | 0.0246 | -0.003 | 0.2602 |
| 322 | 0.3089 | 0.0228 | -0.003 | 0.2731 |
| 324 | 0.3252 | 0.0215 | -0.003 | 0.2867 |
| 326 | 0.3423 | 0.0193 | -0.003 | 0.3004 |
| 328 | 0.3594 | 0.0181 | -0.003 | 0.3149 |
| 330 | 0.3764 | 0.0172 | -0.003 | 0.3304 |
| 332 | 0.3944 | 0.0159 | -0.003 | 0.3458 |
| 334 | 0.4107 | 0.0152 | -0.003 | 0.3604 |
| 336 | 0.4268 | 0.0151 | -0.003 | 0.3739 |
| 338 | 0.4412 | 0.014 | -0.003 | 0.3871 |
| 340 | 0.4545 | 0.0139 | -0.003 | 0.3992 |
| 342 | 0.4663 | 0.0135 | -0.002 | 0.4094 |
| 344 | 0.4755 | 0.0134 | -0.003 | 0.4183 |
| 346 | 0.4842 | 0.013 | -0.002 | 0.4261 |
| 348 | 0.4914 | 0.013 | -0.003 | 0.432 |
| 350 | 0.495 | 0.0129 | -0.001 | 0.4363 |
| 352 | 0.4971 | 0.0125 | -0.002 | 0.4392 |
| 354 | 0.4973 | 0.0125 | -0.002 | 0.4397 |
| 356 | 0.4949 | 0.0127 | -0.002 | 0.4378 |
| 358 | 0.4905 | 0.0122 | -0.002 | 0.4339 |
| 360 | 0.4841 | 0.0127 | -0.002 | 0.4281 |
| 362 | 0.4755 | 0.0126 | -0.001 | 0.4207 |
| 364 | 0.4642 | 0.0122 | -0.002 | 0.4115 |
| 366 | 0.4519 | 0.0124 | -0.001 | 0.4005 |
| 368 | 0.4366 | 0.0124 | -0.001 | 0.3872 |
| 370 | 0.4212 | 0.0123 | -0.001 | 0.3733 |
| 372 | 0.4032 | 0.012 | -0.001 | 0.3579 |
| 374 | 0.3847 | 0.0117 | -0.001 | 0.3407 |
| 376 | 0.365 | 0.0114 | -0.001 | 0.3232 |
| 378 | 0.3445 | 0.0118 | 0 | 0.305 |
| 380 | 0.3236 | 0.012 | -0.001 | 0.2866 |
| 382 | 0.3038 | 0.0114 | 0 | 0.2662 |
| 384 | 0.2815 | 0.011 | 0 | 0.2484 |
| 386 | 0.2556 | 0.0103 | -0.002 | 0.2245 |
| 388 | 0.2349 | 0.0111 | 0 | 0.2061 |
| 390 | 0.2136 | 0.0109 | 0 | 0.1865 |
| 392 | 0.1929 | 0.0108 | 0 | 0.1678 |
| 394 | 0.173 | 0.0107 | 0 | 0.1496 |
| 396 | 0.1545 | 0.0105 | 0 | 0.1327 |
| 398 | 0.1365 | 0.0105 | 0 | 0.1164 |
| 400 | 0.1198 | 0.0106 | 0 | 0.101 |
| 402 | 0.104 | 0.0103 | 0 | 0.0866 |
| 404 | 0.0897 | 0.0103 | 0 | 0.0735 |
| 406 | 0.0771 | 0.0104 | 0 | 0.0617 |
| 408 | 0.0655 | 0.0102 | 0 | 0.0507 |
| 410 | 0.0547 | 0.0101 | 0.0001 | 0.0408 |
| 412 | 0.0454 | 0.01 | 0.0003 | 0.032 |
| 414 | 0.0374 | 0.0101 | 0.0003 | 0.0246 |
| 416 | 0.0305 | 0.0098 | 0.0003 | 0.0182 |
| 418 | 0.0248 | 0.0098 | 0.0004 | 0.0128 |
| 420 | 0.0202 | 0.0097 | 0.0006 | 0.0086 |
| 422 | 0.0165 | 0.0098 | 0.0008 | 0.0052 |
| 424 | 0.0138 | 0.0098 | 0.0007 | 0.0027 |
| 426 | 0.0117 | 0.0098 | 0.0009 | 0.0009 |
| 428 | 0.0102 | 0.0096 | 0.0009 | 0 |
| 430 | 0.0089 | 0.0095 | 0.001 | -0.001 |
| 432 | 0.0081 | 0.0097 | 0.001 | -0.002 |
| 434 | 0.0077 | 0.0096 | 0.0009 | -0.002 |
| 436 | 0.0072 | 0.0095 | 0.0012 | -0.003 |
| 438 | 0.0069 | 0.0096 | 0.0011 | -0.003 |

Fig. S1. UV absorption spectra of standard solutions of 50 µg/ mL OXA, 50 µg /mL 5-FU and 10 µg /mL LU and their mixture in deionized water

D1

| 𝜆 (nm) | MIX | OXA | 5-FU | LU |
| --- | --- | --- | --- | --- |
| 200 | 1.0559 | -0.0505 | 1.2319 | -0.1282 |
| 202 | 0.5345 | -0.2758 | 0.286 | -0.1568 |
| 204 | -0.1884 | -0.2947 | -0.2902 | -0.1189 |
| 206 | -0.3903 | -0.333 | -0.7029 | -0.0924 |
| 208 | -0.798 | -0.3728 | -1.0064 | -0.0763 |
| 210 | -1.1342 | -0.3954 | -1.2885 | -0.0679 |
| 212 | -1.5496 | -0.3948 | -1.4869 | -0.0668 |
| 214 | -1.693 | -0.374 | -1.5585 | -0.0714 |
| 216 | -1.7061 | -0.3373 | -1.486 | -0.0763 |
| 218 | -1.5597 | -0.2912 | -1.3129 | -0.0795 |
| 220 | -1.3043 | -0.241 | -1.0539 | -0.0813 |
| 222 | -0.9733 | -0.193 | -0.7522 | -0.0823 |
| 224 | -0.6364 | -0.1514 | -0.4427 | -0.0822 |
| 226 | -0.3305 | -0.1172 | -0.1609 | -0.0807 |
| 228 | -0.0595 | -0.0893 | 0.0887 | -0.0777 |
| 230 | 0.1752 | -0.0667 | 0.3039 | -0.0715 |
| 232 | 0.3685 | -0.0485 | 0.4801 | -0.0632 |
| 234 | 0.5224 | -0.0346 | 0.6162 | -0.0534 |
| 236 | 0.6432 | -0.0248 | 0.7192 | -0.0435 |
| 238 | 0.7371 | -0.0176 | 0.8005 | -0.0334 |
| 240 | 0.8092 | -0.0128 | 0.8606 | -0.0246 |
| 242 | 0.8599 | -0.0103 | 0.8963 | -0.0165 |
| 244 | 0.8735 | -0.0087 | 0.9063 | -0.01 |
| 246 | 0.8599 | -0.008 | 0.8823 | -0.0048 |
| 248 | 0.8066 | -0.0081 | 0.8279 | -0.0002 |
| 250 | 0.7369 | -0.0082 | 0.7417 | 0.0029 |
| 252 | 0.6108 | -0.0087 | 0.6232 | 0.0048 |
| 254 | 0.4535 | -0.0098 | 0.4745 | 0.0054 |
| 256 | 0.2645 | -0.0106 | 0.3463 | 0.0056 |
| 258 | 0.1644 | -0.0118 | 0.1896 | 0.0054 |
| 260 | -0.0185 | -0.0125 | 0.0272 | 0.0061 |
| 262 | -0.1846 | -0.0131 | -0.1364 | 0.0073 |
| 264 | -0.332 | -0.0135 | -0.2926 | 0.01 |
| 266 | -0.4606 | -0.0146 | -0.4829 | 0.0131 |
| 268 | -0.6826 | -0.0143 | -0.6568 | 0.0163 |
| 270 | -0.8537 | -0.0142 | -0.8304 | 0.0183 |
| 272 | -0.9878 | -0.0134 | -0.9805 | 0.0186 |
| 274 | -1.0994 | -0.0131 | -1.0955 | 0.015 |
| 276 | -1.1837 | -0.0121 | -1.1709 | 0.0088 |
| 278 | -1.2201 | -0.0117 | -1.2011 | -0.0009 |
| 280 | -1.1873 | -0.0118 | -1.2422 | -0.0122 |
| 282 | -1.1136 | -0.0118 | -1.2028 | -0.0245 |
| 284 | -0.9936 | -0.011 | -1.09 | -0.0356 |
| 286 | -1 | -0.0106 | -1 | -0.0464 |
| 288 | -0.6686 | -0.0106 | -0.799 | -0.0536 |
| 290 | -0.5001 | -0.0101 | -0.58 | -0.0564 |
| 292 | -0.3539 | -0.01 | -0.41 | -0.0538 |
| 294 | -0.2342 | -0.0102 | -0.233 | -0.0462 |
| 296 | -0.1405 | -0.0099 | -0.1112 | -0.033 |
| 298 | -0.0719 | -0.009 | -0.0598 | -0.0173 |
| 300 | -0.0248 | -0.009 | -0.0318 | -0.0017 |
| 302 | 0.0071 | -0.0086 | -0.0156 | 0.0127 |
| 304 | 0.0292 | -0.0083 | -0.0072 | 0.0249 |
| 306 | 0.0455 | -0.0081 | -0.0031 | 0.0353 |
| 308 | 0.0568 | -0.0085 | -0.002 | 0.0435 |
| 310 | 0.0649 | -0.0084 | 0 | 0.05 |
| 312 | 0.0717 | -0.0086 | 0 | 0.0553 |
| 314 | 0.0766 | -0.0082 | 0 | 0.0602 |
| 316 | 0.0799 | -0.0089 | 0 | 0.0634 |
| 318 | 0.0823 | -0.0084 | 0 | 0.0665 |
| 320 | 0.0841 | -0.0074 | 0 | 0.0702 |
| 322 | 0.0855 | -0.0069 | 0 | 0.0727 |
| 324 | 0.0855 | -0.0063 | 0 | 0.0737 |
| 326 | 0.0845 | -0.0042 | 0 | 0.0735 |
| 328 | 0.0818 | -0.0041 | 0 | 0.0722 |
| 330 | 0.0781 | -0.0033 | 0 | 0.0688 |
| 332 | 0.0719 | -0.0024 | 0.001 | 0.0636 |
| 334 | 0.0648 | -0.0018 | 0 | 0.0579 |
| 336 | 0.0574 | -0.0021 | 0.001 | 0.0522 |
| 338 | 0.0502 | -0.001 | 0 | 0.0449 |
| 340 | 0.0405 | -0.001 | 0.002 | 0.0371 |
| 342 | 0.0308 | -0.001 | 0 | 0.0298 |
| 344 | 0.0218 | -0.0009 | 0.001 | 0.0214 |
| 346 | 0.0107 | -0.0003 | 0 | 0.0117 |
| 348 | -0.0009 | -0.0008 | 0.001 | 0.0019 |
| 350 | -0.0109 | -0.0002 | -0.001 | -0.0082 |
| 352 | -0.0216 | 0.0001 | 0.001 | -0.0185 |
| 354 | -0.0331 | -0.0003 | 0 | -0.0282 |
| 356 | -0.043 | -0.0003 | 0.001 | -0.0373 |
| 358 | -0.0539 | 0.0002 | 0.001 | -0.0467 |
| 360 | -0.0629 | -0.0004 | 0.001 | -0.0548 |
| 362 | -0.0723 | -0.0006 | 0 | -0.0628 |
| 364 | -0.0795 | -0.0005 | 0.001 | -0.0708 |
| 366 | -0.0869 | -0.001 | 0 | -0.0773 |
| 368 | -0.0921 | -0.0006 | 0.001 | -0.0822 |
| 370 | -0.0976 | -0.0003 | 0 | -0.0867 |
| 372 | -0.0994 | -0.0006 | 0.001 | -0.0917 |
| 374 | -0.1032 | -0.0007 | 0.001 | -0.0923 |
| 376 | -0.1094 | -0.0011 | -0.001 | -0.0987 |
| 378 | -0.1096 | -0.0007 | 0 | -0.0989 |
| 380 | -0.11 | -0.0011 | 0.001 | -0.1001 |
| 382 | -0.1109 | -0.0006 | 0 | -0.0984 |
| 384 | -0.1085 | -0.0003 | 0 | -0.0988 |
| 386 | -0.1011 | 0.0002 | 0.002 | -0.0918 |
| 388 | -0.0984 | -0.0006 | 0 | -0.0897 |
| 390 | -0.0938 | -0.0003 | 0 | -0.0855 |
| 392 | -0.0889 | -0.0005 | 0 | -0.0812 |
| 394 | -0.0833 | -0.0004 | 0 | -0.0761 |
| 396 | -0.0774 | -0.0001 | 0 | -0.071 |
| 398 | -0.071 | -0.0003 | 0 | -0.0657 |
| 400 | -0.0651 | -0.0005 | 0.0001 | -0.0602 |
| 402 | -0.0586 | -0.0003 | 0.0003 | -0.0546 |
| 404 | -0.0523 | -0.0002 | 0.0003 | -0.0489 |
| 406 | -0.0466 | -0.0006 | 0.0003 | -0.0435 |
| 408 | -0.0407 | -0.0004 | 0.0004 | -0.0379 |
| 410 | -0.0345 | -0.0004 | 0.0005 | -0.0322 |
| 412 | -0.0289 | -0.0002 | 0.0005 | -0.0268 |
| 414 | -0.0236 | -0.0003 | 0.0004 | -0.0219 |
| 416 | -0.0188 | 0 | 0.0006 | -0.0173 |
| 418 | -0.0146 | -0.0002 | 0.0005 | -0.0128 |
| 420 | -0.0113 | -0.0002 | 0.0004 | -0.0096 |
| 422 | -0.0084 | -0.0001 | 0.0002 | -0.0072 |
| 424 | -0.0061 | -0.0002 | 0.0002 | -0.0047 |
| 426 | -0.0045 | -0.0003 | 0.0003 | -0.0039 |
| 428 | -0.0033 | 0 | 0.0002 | -0.003 |

Fig.S2. First derivative spectra of standard solutions of 50 µg/ mL OXA, 50 µg /mL 5-FU ,and 10 µg /mL LU and their mixture in deionized water (∆ $\lambda$ =10 nm)

Table S1. Robustness evaluation of the proposed spectrophotometric methods for OXA, 5-FU, and LU under deliberate operational variations (n=3)

|  | | Mean %recovery ± RSD (%) | | | | | |
| --- | --- | --- | --- | --- | --- | --- | --- |
| Experimental Parameter | Variation Level | OXA | 5-FU | 5-FU | LU | LU | LU |
|  |  | HDDR | D1 | HDDR | D0 | D1 | HDDR |
| Wavelength Setting (λ) | Nominal (0 nm) | 99.85 ± 0.65 | 100.12 ± 0.42 | 100.30 ± 0.35 | 99.90 ± 0.78 | 100.15 ± 0.50 | 99.38 ± 0.97 |
|  | -1.0 nm | 99.40 ± 0.88 | 99.75 ± 0.54 | 99.80 ± 0.62 | 99.55 ± 0.92 | 99.70 ± 0.70 | 99.10 ± 1.05 |
|  | +1.0 nm | 100.20 ± 0.72 | 100.35 ± 0.60 | 100.45 ± 0.48 | 100.15 ± 0.81 | 100.50 ± 0.65 | 99.70 ± 0.85 |
| Divisor Concentration | Nominal (0%) | 99.85 ± 0.65 | 100.12 ± 0.42 | 100.30 ± 0.35 | 99.90 ± 0.78 | 100.15 ± 0.50 | 99.38 ± 0.97 |
|  | -2.0 % | 99.52 ± 0.95 | 99.68 ± 0.48 | 99.95 ± 0.55 | 99.60 ± 1.00 | 99.85 ± 0.82 | 99.05 ± 1.12 |
|  | +2.0 % | 100.10 ± 0.81 | 100.28 ± 0.55 | 100.48 ± 0.42 | 100.25 ± 1.05 | 100.65± 0.60 | 99.65 ± 0.90 |

Mean %recovery ± RSD (%) :Mean ± Relative standard deviation %

**Table S2. Quantitative comparison between the proposed HDDR method and reported HPLC methods^11,12^**

| Parameter | The reported HPLC-DAD method [11] | The reported HPLC-DAD method [12] | The Proposed HDDR |
| --- | --- | --- | --- |
| Analysis time (min/sample) | 68 | 20 | <1 ( direct measurement ) |
| Physical separation | Required | Required | Not required |
| Organic solvent consumption | High | High | None (water) |
| Sample throughput (samples/ h) | 0.88 | ~3 | >30 |

| The proposed UV spectrophotometric method | | The reported  HPLC-DAD method [11] | | The reported  HPLC-DAD method [12] | |
| --- | --- | --- | --- | --- | --- |
| Reagents  Deionized water | Penalty point | Reagents  Methanol  Trifluoroacetic acid  water | Penalty point | Reagents  Methanol  Trifluoroacetic acid  water | Penalty point |
|  | 0  0  ∑1 |  | 1X6=6  1x4=4  0  ∑10 |  | 1X6=6  1x4=4  0  ∑10 |
| Instrument  Energy  Occupational hazard  waste | 1  0  3  ∑4 | Instrument  Energy  Occupational hazard  waste | 1  0  5  ∑6 | Instrument  Energy  Occupational hazard  waste | 1  0  5  ∑6 |
| Total penalty points | 4 | Total penalty points | 16 | Total penalty points | 16 |
| Eco scale assessment score | 100-4= 96 | Eco scale assessment score | 100-16=84 | Eco scale assessment score | 100-16=84 |

Table S3. Details of evaluation of the degree of greenness of the proposed spectrophotometric method

in comparison with the reported HPLC-DAD methods^11,12^	using AES

| Method | RGB Profile & Scores | Justification & Key Remarks |
| --- | --- | --- |
| The proposed UV spectrophotometric method |  | Redness (96.3%): High accuracy, precision, and application scope without requiring chromatographic separation.  Greenness (100.0%): Zero toxic organic solvents, minimal energy consumption, and virtually no waste generation.    Blueness (99.4%): Exceptional cost and time efficiency with simple, routine instrumentation.  Whiteness (98.5%): Highly balanced performance combining optimal eco-friendliness with practical utility. |
| reported  HPLC-DAD method [11] |  | Redness (97.5%): High sensitivity and precision owing to chromatographic separation capabilities.  Greenness (70.8%): Lower eco-friendliness due to hazardous organic mobile phases and energy usage.  Blueness (84.4%): Higher operational costs, longer run times, and specialized instrument requirements.  Whiteness (84.2%): Compromised overall balance due to environmental and economic penalties. |
| reported  HPLC-DAD method [12] |  | Redness (100.0%): Excellent analytical performance with wide scope and high sensitivity.  Greenness (82.1%): Moderate greenness penalty from solvent consumption and organic waste.  Blueness (91.9%): Improved operational efficiency over [11] but still constrained by HPLC run times and solvent costs.  Whiteness (91.3%): Good performance overall, but lower than the proposed UV method in sustainability |

Table S4**.** Comparison of the multi-colored analytical profiles (WAC/RGB principles) and quantitative scoring justification for the proposed UV spectrophotometric method versus reported HPLC methods^11,12^	.
